# How artificial intelligence can enable personalized mesenchymal stem cell–based therapeutic strategies in systemic lupus erythematosus

**DOI:** 10.3389/fimmu.2025.1654117

**Published:** 2025-11-26

**Authors:** Sushmitha Rajeev Kumar, Khor Kai He, Yogeswaran Lokanathan, Anand Gaurav, Khatijah Yusoff, M. Fatima Macedo, Subha Bhassu

**Affiliations:** 1 Malaysia Genome and Vaccine Institute, National Institute of Biotechnology Malaysia, Selangor, Malaysia; 2 Animal Genetics and Genome Evolutionary Biology Laboratory, Division of Microbiology and Molecular Genetics, Institute of Biological Sciences, Faculty of Science, University of Malaya, Kuala Lumpur, Malaysia; 3 Department of Tissue Engineering and Regenerative Medicine (DTERM), Faculty of Medicine, University Kebangsaan Malaysia, Kuala Lumpur, Malaysia; 4 Department of Pharmaceutical Sciences, School of Health Science and Technology, University of Petroleum and Energy Studies (UPES), Dehradun, Uttarakhand, India; 5 Algarve Biomedical Center Research Institute (ABC-RI), Faculdade de Medicina e Ciências Biomédicas, Universidade do Algarve (UAlg), Faro, Portugal; 6 Instituto de Investigação e Inovação em Saúde (i3S), Universidade do Porto, Porto, Portugal

**Keywords:** machine learning, stem cells, lupus, genetic modifications, personalised medicine, precision therapeutics

## Abstract

Mesenchymal Stromal Cells (MSCs) are increasingly recognized as promising candidates for treating Systemic Lupus Erythematosus (SLE) due to their immunomodulatory and regenerative properties. However, their therapeutic efficacy remains inconsistent, largely due to the heterogeneity of MSC origins, culture conditions, cell quality, host immune interactions, and the influence of immunosuppressive treatments. Artificial Intelligence (AI) offers powerful tools to address these challenges by optimising MSC modification and application. This review explores how AI can identify optimal genetic and epigenetic targets, predict MSC behaviour under different environmental and priming conditions, and design personalise therapies tailored to individual patients. Moreover, AI enables the analysis of extensive datasets to refine dosing strategies and improve the integration of MSC therapy with immunosuppressants. By enhancing the precision, consistency, and personalisation of MSC-based interventions, AI has the potential to significantly improve therapeutic outcomes in SLE, advancing the field toward more effective and patient-centred autoimmune disease management.

## Introduction

Systemic lupus erythematosus (SLE) is a chronic autoimmune disorder that affects the body multi-systematically, with varying clinical manifestations depending on the patient (1). The global prevalence of SLE is estimated to affect approximately 3.17 million adults worldwide and has been increasing over time (2). Common visual clinical representation involves musculoskeletal and dermatological manifestations, which include fever, joint pains, and the hallmark “malar rash” that appears across the cheeks and nasal bridge (3). The severity of symptoms evolves with time, showing manifestations affecting functionality in multiple organs such as renal, neurological, pulmonary, gastrointestinal, cardiovascular, and more (3). SLE places a significant burden on patients’ quality of life, affecting both physical and mental health (4). The multisystemic involvement showcases the importance of SLE, as it poses significant risks for both morbidity and mortality, being up to 5 times more likely to die compared to the general population (5).

SLE is most prevalent in women aged between adolescence and menopause, with a female predominance of 9:1 (6–8). The heightened susceptibility in women is driven by a multitude of factors, including sex hormones (such as oestrogen and prolactin), which promote autoimmunity and trigger B cell activation and secretion of autoantibodies (9). Oestrogen (especially 17β-oestradiol (E2/oestrogen)) react with many immune cell types such as macrophages, mast cells, dendritic cells (DCs), T cells and B cells by reacting with either oestrogen receptor α (ERα) or Erβ expressed by these cells. Rapid responses are then initiated as part of lipid signalling rafts. Oestrogen also promotes the activation, survival, hypermutation, and class switch recombination in B cells, which causes higher antibody/autoantibody responses in females (9). Additionally, the higher prevalence of SLE in women has been linked to genetic factors related to the X chromosome. Specific X-linked genes such as TLR7, IRAK2 and MECP2 further support the role of the X chromosome in SLE susceptibility (10). Some X-linked genes, like TLR7, can escape X-inactivation (XCI) in certain immune cells, leading to biallelic gene expression that further contributes to disease susceptibility (11).

## Immune dysregulation in SLE

The pathogenesis of SLE involves the dysregulation of both innate and adaptive immune responses, leading to pathogenic autoantibodies production, B cell hyperactivation, cytokine imbalance, and ultimately tissue and organ damage (6). One key initiating event is the defective clearance of apoptotic cells, caused by impaired phagocytosis or complement deficiencies. This results in the accumulation of apoptotic debris and exposure of nuclear self- antigens such as double-stranded DNA (dsDNA) to the immune system (12). Autoreactive B cells recognise these nuclear antigens and, with the help of autoreactive T helper cells, undergo clonal expansion and differentiate into plasma cells, that secrete high-affinity autoantibodies. These antibodies form immune complexes (ICs) that deposit in various tissues, including the kidneys, joints, skin, and brain. Once deposited, ICs activate complement and engage Fcγ receptors, triggering recruitment of neutrophils, macrophages, and dendritic cells (DCs) (6). Plasmacytoid dendritic cells (pDCs) subsequently produce large amounts of type I interferons, especially IFN-α, which amplify B and T cell activation in a self-perpetuating cycle.

Innate immune cells further contribute to this dysregulation. Neutrophils and DCs detect apoptotic debris and ICs via Toll-like receptors (TLRs), initiating inflammatory cascades characterised by type I interferon release (13, 14). IFN-α plays a pivotal role by breaking immune tolerance: it activates antigen-presenting cells, enhances MHC class I and II expression, and upregulates co-stimulatory molecules (CD80, CD86, and CD40), thereby promoting autoreactive T-cell activation (12, 15, 16). IFN-α also induces NETosis in neutrophils, releasing neutrophil extracellular traps (NETs) that expose further autoantigens, perpetuating the autoimmune cycle (17).

Beyond antigen presentation, IFN-α fosters a pro-inflammatory environment by stimulating cytokine production. IL-12 promotes Th1 differentiation, while IL-6 and IL-23 drive Th17 differentiation (18). The resulting Th1 and Th17 cells secrete IFN-γ and IL-17, amplifying inflammation, whereas regulatory T cell (Tregs) function is impaired through downregulation of FOXP3, weakening immune suppression (19).

On the adaptive side, B cells are hyperactivated via B-cell receptor (BCR) signalling and interactions with T follicular helper (Tfh) cells, producing autoantibodies against dsDNA and other nuclear antigens (20). These autoantibodies form pathogenic ICs that deposit in tissues, activating complement and driving chronic inflammation and organ damage (21). As disease progresses, a network of pro-inflammatory cytokines, including IL-1, IL-6, TNF-α, and IL-17, reinforces persistent immune activation and tissue damage.

## Genetic and environmental factors contributing to SLE

The exact cause of SLE remains unclear, but both genetic predisposition and environmental triggers are recognised as critical contributors to its pathogenesis. Among genetic factors, strong associations have been identified with the MHC, particularly the MHC class II region. Variants in genes regulating immune responses like C1q, C2, C4, Fcγ receptors, and signalling molecules involved in type I interferon pathways, have also been linked to increased susceptibility. Furthermore, the markedly higher prevalence of SLE in women suggests a role for X chromosome-linked genetic factors and hormonal influences, especially oestrogen, in disease susceptibility (10).

Environmental factors play an equally important role in triggering disease onset or flares in genetically predisposed individuals. Viral infections, particularly Epstein-Barr Virus (EBV), have been implicated through mechanisms such as molecular mimicry, where viral proteins resemble host antigens, leading to a breakdown of immune tolerance and autoimmunity (22). Ultraviolet (UV) radiation, especially UV-B rays, exacerbates disease activity by inducing keratinocyte apoptosis and exposing nuclear autoantigens, often resulting in cutaneous manifestations such as malar rash. Drugs such as hydralazine, procainamide, and isoniazid are well documented causes of drug-induced lupus, which shares clinical features with idiopathic SLE but usually resolves upon drug withdrawals (8, 23).

Other contributing factors include smoking, which enhances oxidative stress and immune activation, and hormonal influences, where oestrogen promotes B-cell hyperactivity and autoantibody production, further explaining the female predominance of SLE. In addition, dysbiosis of the gut microbiome has emerged as a potential environmental factor, with alterations in microbial composition shown to influence immune regulation and autoimmunity.

Together, the interplay of genetic predisposition, hormonal influences, and environmental triggers such as infections, UV radiation, and drugs creates a multifactorial foundation for SLE development and progression.

## Limitations of existing treatments for SLE

Current treatments of SLE involve the use of general immunosuppressants and inhibitors that are designed to affect different pathways involved in SLE pathogenesis to reduce inflammation by inhibiting the pro-inflammatory cytokine signalling, TLR activation, and T-cell proliferation (24). However, these treatments come with serious side effects, which are recorded in **Tables 1A**, **B**. Apart from the side effects, the failure of existing treatment is also caused by significant drug resistance to therapeutics over time. SLE patients tend to develop resistance against medications such as corticosteroids (42).

**Table 1A:** Conventional immunosuppressants and corticosteroids used in SLE.

Immunosuppressant/Corticosteroid therapy	Mechanism of action	Side effects	References
Azathioprine (AZA)	A purine analog that turns into active metabolites to block purine synthesis and stop DNA replication, leading to immunosuppressive effects.	Nausea, fever, fatigue, arthralgias/myalgia, rash, bone marrow suppression	(25)
Cyclosporine (CsA)	Suppress cell-mediated immune reaction while inhibiting synthesis of interleukins (IL).	Hypertension, arrhythmia, convulsions, renal issues, dyslipidemia, malignant lymphomas	(26)
Cyclophosphamide (CYC)	Antimitotic, antineoplastic, and immunosuppressive effects selective to T cells. Lowers the secretion of IL-12 and increases secretion of IL-4 and IL-10.	Gonadal and bladder toxicity, vomiting, nausea, alopecia, haemorrhagic cystitis.	(27)
Voclosporin	Calcineurin inhibitor to manage lupus nephritis. Inhibit production of IL-2 and prevent proliferation of effector T cells.	Acute and chronic nephrotoxicity, reduced glomerular filtration rate, hypertension, neurotoxicity, liver injury with jaundice.	(28)
Tacrolimus	Inhibits the proliferation of T cells via the calcineurin inhibitor.	Hypertension, arrhythmias, headaches, insomnia, angina pectoris, acne vulgaris, alopecia, rash, weight gain, nausea, vomiting, diarrhoea.	(29)
Methotrexate	Reduces the activation of T-cells, diminishes B-cell responses, promotes the activation of CD95-positive T cells, and interferes with the interaction of interleukin β1.	Nausea, vomiting, loss of appetite, mucosal ulcers hepatotoxicity, and potential of teratogenesis in females of the child-bearing group.	(30)
Mycophenolate mofetil (MMF)	Lowers antibody synthesis and limits the expansion of both T and B lymphocyte populations.	Nausea, diarrhoea, leukopenia, urinary tract infection, renal flare, urticaria and myopathy	(31)
Hydroxychloroquine (HCQ)	Inhibit toll-like receptors (TLRs), enzymes, NK cells, and cytokine release. Involvement in T cell polarisation and apoptosis.	Cardiomyopathy, dizziness, fatigue, cytopenia, hyperpigmentation	(32)
Glucocorticoids (GCs)	Inhibits B and T cells and phagocytes. Activates the cytosolic GC receptor (cGCR) that suppresses pro-inflammatory cytokines and upregulates anti-inflammatory cytokines.	Ecchymosis, cutaneous thinning and atrophy, acneiform eruptions, mild hirsutism, facial erythema, striae, increased body weight, delayed wound healing, hair thinning, and perioral dermatitis.	(33, 34)

**Table 1B:** Biologics and targeted small-molecular inhibitors for SLE.

Biologic/Small molecular inhibitor therapy	Mechanism of action	Side effects	References
Belimumab	Inhibits B lymphocyte stimulator protein and downregulates B cell activity.	Infection, infusion reactions, hypersensitivity, headache, nausea and fatigue.	(35)
Antifrolumab	Inhibits the formation of IFN complexes and gene transcription.	Cough, trouble breathing, cold symptoms	(36)
Rituximab	Affects the functionality of B cells and decreases plasma cell production	Allergies caused by infusion reactions, infections, skin rash, alopecia, respiratory and cardiovascular effects	(37)
Janus Kinases inhibitors	Suppresses cytokine production that is involved in Th1, Th2, Th17 and Th22 providing anti-inflammatory effects	Upper respiratory infections, headaches, nausea, acne, urinary tract infections, gastrointestinal	(38)
Bruton’s tyrosine kinase inhibitors	Hinders the activity of BTK protein that is involved in B cells maturation and activation.	Haemorrhage, hypertension, pneumonia, infection, contusion, nausea, fatigue, arthralgia	(39)
Proteasome inhibitors	Depletes plasma cells and inhibits type-1 IFN activity	Infections, hypogammaglobulinemia	(40, 41)

Conventional drugs form the backbone of SLE management and remain widely used due to their accessibility and broad immunosuppressive effects. However, while they are effective in controlling SLE, they lack specificity, and cause toxicity accumulation, resulting in adverse side effects like bone marrow suppression, gonadal toxicity, organ damage and teratogenicity. This significantly limits long-term use. Biologics and targeted inhibitors selectively block key immune pathways, potentially halting symptoms that result from the dysregulation of the specific pathway. This offers more precise treatment, and improved disease control. However, these treatments often resulted in adverse events, such as infection, infusion reactions, as well as cardiovascular and metabolic risks. They are also only controlling the disease without curing it, which underscores the therapeutic gap.

The limitations of these existing treatments have prompted the exploration of novel treatment strategies to improve the disease management and long-term outcomes. Current research is focused on a variety of approaches, ranging from immunotherapies to advanced cell-based therapies, such as hematopoietic stem cells (HSCs), MSCs, CAR T cells. Increasing attention has also been directed toward MSC-derived extracellular vesicle (EV) therapy, including molecular modifications of EVs to selectively upregulate or downregulate microRNAs implicated in SLE pathways. These emerging strategies hold promise as more effective treatments with reduced clinical side effects compared to conventional options.

Immunotherapies under investigation include antibody-based therapies such as B cell-targeted agents, CD40-CD40L interaction-targeted inhibitors, CD38-targeted therapies, and cytokine-targeted interventions (43, 44). While many of these approaches show potential, the heterogeneity of SLE continues to pose significant challenges in developing broadly effective treatments (44).

Beyond antibody- and cytokine-based strategies, cell-based therapies are gaining momentum in SLE treatment. Hematopoietic stem cell transplantation (HSCT) can improve immune tolerance by eliminating autoreactive CD27+ memory cells and restructuring adaptive immunity (45). However, HSCT is generally reserved for patients with severe therapy resistant SLE due to its high risk of transplant-related mortality, infections, and adverse effects such as allergies, bone pain and heart failure (46).

CAR T cell-therapy, which involves genetic modification of T cells to target pathogenic B-cells, has also shown promise. Nevertheless, it faces significant challenges, including high treatment costs and the risk of severe adverse events such as cytokine release syndrome (CRS), hemophagocytic lymphohistiocytosis/macrophage activation syndrome (HLH/MAS), and immune effector cell-associated neurotoxicity syndrome (ICANS) (47, 48). Moreover, current CAR-T technologies are typically designed to target specific antigens or pathways, which may not fully capture the complexity of SLE pathogenesis (1, 49).

In parallel, MSC-derived EVs are emerging as a promising therapeutic avenue, with growing evidence supporting their immunomodulatory and regenerative potential in SLE (50). Given that this area has been comprehensively reviewed elsewhere (50), it will not be elaborated further here.

## Mesenchymal stromal cells and their therapeutic relevance in SLE

MSCs are multipotent cells of perivascular origin with regenerative and immunomodulatory potential, making them attractive candidates for novel therapies in SLE. This disease is characterized by immune dysregulation and progressive organ damage, where MSCs can offer a means to recalibrate the immune system. Through the release of anti-inflammatory cytokines and immunoregulatory molecules, MSCs can suppress hyperactive immune responses and mitigate tissue injury. Their dual regenerative and immunosuppressive capabilities highlight their promise as a long-term therapeutic strategy.

MSCs are defined by expression of cellular markers such as CD73, CD105, and CD90 (50), while lacking hematopoietic markers like CD14, CD34, and CD45 (51). Additional markers, including CD10, CD13, CD44, and CD146 (51) may vary with tissue origin. However, MSC populations are inherently heterogeneous, with behaviour influenced by on the surrounding microenvironment (52). This variability contributes to inconsistent therapeutic outcomes in clinical applications. AI-driven single-cell profiling and predictive modelling can help classify functional MSC subpopulations, standardize quality, and predict therapeutic potency across diverse patient contexts.

A defining characteristic of MSCs is their tri-lineage differentiation potential, enabling them to form bone, cartilage, and adipose tissues. Beyond musculoskeletal repair, their paracrine effects contribute to cardiac and immune tissue recovery (50, 52, 53). Among adult MSCs, Wharton’s Jelly-MSCs (WJ-MSCs) demonstrate higher bone marrow-derived MSCs, which remain the most widely tested in clinical trials (51). In addition to differentiation, MSCs exhibit self-renewal, long-term proliferation, and extensive paracrine signalling. These properties are central not only to tissue regeneration but also to the immunoregulation required for SLE therapy. Here, AI-guided modelling of differentiation pathways and culture conditions can optimise expansion protocols, predict senescence, and enhance reproducibility for clinical-grade MSC production (50, 51).

### Toward optimized MSC-based therapy for SLE

The therapeutic potential of MSCs lies in their ability to both restore tissue integrity and rebalance immune homeostasis. However, variability in MSC sources, culture conditions, and patient responses remains a barrier to clinical success. By integrating AI-based analytics with molecular and clinical datasets, researchers can identify optimal MSC phenotypes, predict immunomodulatory performance, and personalize treatment strategies for SLE patients. This convergence of cell biology and computational intelligence represents the next frontier in developing consistent, safe, and effective MSC-based therapies.

## Therapeutic potential of MSCs in autoimmune diseases

### Immune evasion and homing ability

MSCs mainly regulate immune responses through multiple mechanisms They promote Th2 differentiation, increase IL-10 secretion, and inhibit pro-inflammatory cytokines such as TNF-α and IFN-γ (51). Additionally, MSCs also suppress dendritic cell maturation, B cell proliferation, and autoantibody production, thereby re-balancing immune function (45, 54). Their immunosuppressive properties is enhanced in inflammatory environments rich in IFN-γ, TNF-α, IL-1α or IL-1β, where they modulate macrophages and neutrophils (55), suppress lymphocyte activity (56), and release prostaglandin E2 (PGE2) to reprogram macrophages toward an anti-inflammatory phenotype (57). Immunoregulation occurs primarily via indoleamine 2, 3-dioxygenase (IDO) in humans and nitric oxide (NO) in mice. Inflammatory cytokines attract immune cells and MSCs to the active sites where IDO or NO are expressed (55).

Their low immunogenicity is mainly due to the absence of MHC class II and co-stimulatory molecules (B7-1, B7–2 and CD40), enabling their use as allogeneic therapies (45, 58). Importantly, they display strong homing ability, migrating to damaged or inflamed tissues, where they recruit regulatory T cells (Tregs) and promote angiogenesis, enhancing both immune control and tissue regeneration (50, 52). These combined properties position MSCs as promising candidates for addressing the chronic inflammation and organ damage central to SLE pathogenesis.

## Implications for SLE therapy

The broad regenerative and immunoregulatory profile of MSCs underscores their potential as an innovative treatment option for autoimmune diseases. For SLE, MSCs offer a dual advantage: controlling the hyperactive immune system while simultaneously repairing damaged tissues. By harnessing these properties, and further refining their application through artificial intelligence to predict potency, personalise dosing, and optimise delivery, MSCs could redefine therapeutic strategies for lupus and related autoimmune disorders.

## Application of MSCs in SLE therapy

### Preclinical and clinical evidence

MSC therapy, alone or in combination with hematopoietic stem cell (HSC) transplantation, has shown encouraging outcomes in both animal models and human patients of SLE. In murine models, these treatments have demonstrated significant improvements, including elevated IL-4 concentrations, improved kidney and liver function, and reduced osteoporosis. Additionally, they also lower levels of anti-ds-DNA antibodies and antinuclear antibodies (ANA) (45).

Therapeutic benefits extend to the reduction of plasma cells, proinflammatory cytokines, and overall disease severity. In lupus nephritis, MSC treatment has been associated with decreased glomerulonephritis, reduced renal protein excretion, lower serum creatinine and albumin levels, improved glomerular filtration rate (GFR), and a significant reduction of anti-dsDNA antibodies (45, 59). Beyond renal manifestations, haematological complications such as leukocytopenia, thrombocytopenia, and anaemia also demonstrate improvement following MSC therapy, largely through the expansion of Tregs.

Together, these findings underscore the potential of MSC- and HSC-based interventions to not only suppress autoimmunity but also restore immune homeostasis and organ function in SLE.

To provide an overview of recent MSCs clinical trials in SLE we summarised key study features, endpoints, and safety findings (**Table 2**). This comparative synthesis highlights both the promise and the variability of MSC interventions. Although there are variabilities in the treatment of some autoimmune diseases with some showing no significant efficacy, SAEs are largely controlled with no direct fatality linked. This shows immense promise, particularly on the unharvested potential of MSCs in the treatment of SLE. There is a total of 4 latest clinical trials with regards to MSCs treatment for SLE. Three clinical trials recorded reduction in SLEDAI score while one recorded no efficacy. In terms of safety, three studies recorded good tolerance to the treatment of MSCs.

**Table 2:** MSC Clinical Trials in the treatment of SLE.

Disease	Study (id/year)	MSC source	Route	n	Key outcomes	Safety	Ref
SLE	NCT01741857 (Multicenter), (2014)	Allogeneic UC-MSCs	IV, 2 doses	40	MCR 33%, improved renal indices, ↓ Lowered SLEDAI scores, steroid taper	Infections, 3 deaths (disease-related); no infusion toxicity	(60)
SLE	NCT00698191 (2010), Phase I	Allogeneic BM-MSCs	IV, single dose	15	↓ Lowered SLEDAI scores, ↓ decreased proteinuria, ↑ increased Treg populations	No treatment-related SAE	(61)
SLE	NCT01741857 / 2016–2019	UC-MSCs	Intravenous infusion	21	MCR 32.5% and PCR 27.5% over 12 months SLEDAI scores decreased	Well-tolerated, no serious side effects	(62)
Lupus nephritis	NCT01539902 (2017), Phase II	Allogeneic UC-MSCs	IV, 4 doses	18	No efficacy *vs* placebo; early futility	2 SAEs in MSC arm (pneumonia, abscess)	(63)

ACR20, American College of Rheumatology 20% improvement criteria; AD-MSCs, adipose-derived mesenchymal stem cells; AE, adverse event; BM-MSCs, bone marrow-derived mesenchymal stem cells; CDAI, Crohn’s Disease Activity Index; EDSS, Expanded Disability Status Scale; IV, intravenous; IT, intrathecal; MCR, major clinical response; PCR, partial clinical response; MSC, mesenchymal stem cell; QoL, quality of life; RA, rheumatoid arthritis; SAE, serious adverse event; SLE, systemic lupus erythematosus; SLEDAI, Systemic Lupus Erythematosus Disease Activity Index; SPMS, secondary progressive multiple sclerosis; SSc, systemic sclerosis; TEAE, treatment-emergent adverse event; Treg, regulatory T cell; UC-MSCs, umbilical cord-derived mesenchymal stem cells.

### Source-dependent mechanisms

Distinct immunomodulatory pathways have been reported for different MSC sources. For treatment of SLE patients using umbilical cord-derived MSCs (UC-MSC), there is upregulation of FLT3L levels, improvement in the number and function of tolerogenic DCs, and a restored balance between Tregs and Th17 cells (62). High levels of TGF-b have also been detected in patients. Moreover, the expression of miR-181a in T cells has been upregulated, further contributing to the immunomodulatory effects of UC-MSC that was recorded in an another ex-vivo mechanistic study (64). In contrast, an ex vivo mechanistic study using bone marrow-derived MSCs (BM-MSCs) has shown suppression of the MEK/ERK signalling pathway and inhibition of peripheral blood mononuclear cell (PBMC) activation. Downregulation of genes such as CD70, ITGAL, selectin-L, and IL-15 has also been observed, further illustrating the therapeutic potential of BM-MSCs in SLE (45).

In SLE, autologous and allogeneic MSCs differ across several key dimensions. Autologous MSCs often show high variability in potency, influenced by patient age, disease activity, prior therapy, and the expansion process (47, 65). Their function may also be compromised by the inflammatory and epigenetic changes in the lupus microenvironment (66, 67). In contrast, allogeneic MSCs, derived from healthy donors, benefit from standardized manufacturing, reduced variability, and are less affected by disease-related priming, which contributes to more consistent outcomes in clinical settings (47, 48).

From a practical standpoint, autologous MSCs require harvesting and culture, delaying treatment (53), while allogeneic products are available off-the-shelf, enabling rapid use in acute cases (53). Immunogenicity is minimal for autologous cells, though functional deficits limit their benefit (54). Allogeneic MSCs carry a low risk of immune reaction due to their immune-privileged nature, though monitoring remains important with repeated dosing (68). Overall, evidence suggests that while autologous MSCs may be suitable in select cases where patient-specific engineering is feasible (69), allogeneic MSCs are generally favoured for SLE due to their reliability, availability, and superior performance in active disease (69).

### Innovation in SLE therapy

Treatment with MSCs has led to improved survival rates in both SLE mice models and human SLE patients, alongside enhancements in renal and liver function (45, 54, 70). A notable decrease in the number of Th17 cells and an increase in Tregs have been observed in both cases. Furthermore, genetically modified MSCs that overexpress IL-37 demonstrate superior immunosuppressive properties compared to standard MSCs or IL-37, suggesting that genetically modified MSCs are more effective in managing SLE.

## Problems associated with the use of MSCs in SLE treatment

### Culture and environment-dependence

The therapeutic potential of MSCs in SLE is strongly influenced by their culture conditions and microenvironment. MSC behaviour is highly plastic, with factors such as substrate stiffness, curvature, biochemical agents, and epigenetic regulation shaping their differentiation and immunomodulatory capacity (52).

### Substrate and materials properties

MSCs cultured on stiffer substrates display enhanced actin-myosin contractility, driving differentiation toward rigid tissue lineages, while softer surfaces help preserve their regenerative and immunomodulatory properties (52). For SLE applications, inappropriate culture conditions may compromise their immunosuppressive functions. Cells cultured in concave environments demonstrate increased motility and reduced stress fibre formation, while those grown on rigid convex surfaces (with a radius of about 500 microns) exhibit flattened nuclei and elongated cell axes, predisposing them toward osteogenic consistency in SLE patients (52). Such unintended lineage priming may reduce therapeutic consistency in SLE patients.

## External agents and biochemical factors

Additives such as ascorbic acid, β-glycerophosphate, vitamin D3, and bone morphogenetic proteins (BMPs) can bias MSCs toward osteogenic differentiation (52). Without careful control, such stimuli may interfere with their capacity to regulate immune responses central to SLE treatment. Epigenetic regulation is critical in determining MSC fate and therapeutic behaviour. Priming strategies, such as pre-exposure to immunomodulatory cytokines (e.g., IL-37), have been shown to enhance MSC efficacy in autoimmune models, including SLE (65, 69). However, the stochastic nature of MSC clonal expansion, exosome production, and variable gene expression adds unpredictability to their therapeutic outcomes.

Interestingly, allogeneic MSCs often outperform autologous cells in SLE patients, showing greater ability to suppress immune hyperactivation and ameliorate disease symptoms (47). This suggests that intrinsic defects in patient-derived MSCs may limit their therapeutic value unless corrected through careful priming or modification.

Overall, these challenges highlight the need for optimised culture systems, controlled priming protocols, and possibly AI-driven predictive modelling to ensure MSCs consistently deliver immunosuppressive and regenerative benefits in SLE therapy.

### Variability in clinical outcomes

While MSC treatments for SLE have shown effectiveness in some studies, they remain ineffective in others. In some cases, no significant improvements were observed in proteinuria, serum albumin, complement levels, Systemic Lupus Erythematosus Disease Activity Index (SLEDAI) score, or renal function when compared to placebo groups in randomised controlled trials (70). Several factors may contribute to these inconsistent outcomes, including the secretion of IL-6, susceptibility to ageing, the unclear aetiology and pathogenesis of SLE, and the complex microenvironment of SLE patients (59).

### Standardisation issues in MSC preparation and SLE aetiology

A major obstacle is the lack of standardised protocols for MSC isolation, expansion, and administration. Differences in production methods, often proprietary or undisclosed, make it difficult to compare studies and interpret outcomes reliably (66). SLE presents generalised symptoms but can vary significantly from patient to patient, making it challenging to identify a uniform treatment. While MSC therapies hold promise, they have yet to offer a complete cure for SLE (59).

## The potential of MSC modifications for SLE therapy

As discussed above, variability in MSC potency, sensitivity to the disease microenvironment, and lack of standardisation limit their therapeutic impact in SLE. To overcome these barriers, a range of strategies has been developed to enhance MSC function and consistency.

### Approaches to modification

Several approaches for MSC modification are available (**Table 3**). Generally, these modifications fall into four categories: 3-dimensional culture of MSCs, priming or pre-treatment of MSCs with various biologics, genetic modification/engineering of MSCs, and the combination therapy of MSCs with immunosuppressants. The detailed breakdown of MSC modifications can be seen in **Supplementary Table 1**, with references.

**Table 3:** Overview of MSC modification strategies for enhancing therapeutic potential.

Modification strategy	Key mechanism	Representative outcomes
3D culture	Spheroid growth enhances cell–cell communication (69)	↑ Increased efficacy in tissue repair and metabolic disease models
Priming (cytokines, biologics, miRNA)	Enhances immunomodulatory and migratory capacity (65, 67)	Improved antibody inhibition (SLE), ↑ migration, tissue regeneration
Genetic engineering	Overexpression of survival/migration genes (e.g., CCR1, CXCR4, IL-37) (68, 71, 72)	↑ Increased MSC lifespan, migration, cytokine secretion, improved outcomes in SLE, osteoarthritis
Combination with drugs	Co-administration with steroids or immunosuppressants (73–75)	Prolonged MSC survival, reduced senescence, superior efficacy *vs* single therapy

### Limitations of MSC modifications

#### Gene modification of MSCs

Genetic modification of MSCs has been explored across multiple disease models, demonstrating the feasibility of enhancing therapeutic traits. While various methods are available for genetic modification, increasing attention has been given to CRISPR technology due to its ease of use, speed, and effectiveness compared to other techniques (76). An increasing number of CRISPR-modified MSCs have progressed to clinical trials (77). **Supplementary Table 2** highlights the breakdown of different approaches used for the genetic modification of MSCs, while **Table 4** below outlines specific methods involving CRISPR/Cas9, along with the modified genes, key findings, and the type of stem cell modified. The application of CRISPR/Cas9 technology has been widely explored across studies using human induced pluripotent stem cells (iPSCs) and embryonic stem cells (ESCs), where genes like SOX2, PAX6, OTX2, and AGO2 were knocked out to examine their roles in developmental processes. These studies demonstrated the feasibility of inducing gene knockouts at any stage of cell differentiation. To simplify, **Table 4** below highlights a simplified breakdown of the various genetic modifications, as well as parameters of consideration.

**Table 4:** Comparative overview of gene-editing nuclease platforms and delivery systems for stem cell modification.

Decision point	Option	Advantages	Limitations / risks	Best suited for…
Nuclease platform	**CRISPR/Cas9**	Rapid design, multiplex editing, broad targetability (76).	Off-target edits, DSB toxicity, possible immune response to Cas9 (78).	Multiplex edits; exploratory targets; AI-guided gRNA design (77).
	**TALENs**	High specificity, good for difficult loci (79).	Complex design, target site constraints (79).	Single precise edits where off-target minimisation is critical (79).
	**ZFNs**	Mature, validated in some contexts (80).	Design complexity, sequence constraints, off-target risk (80).	Use where validated ZFN assets exist (80).
Delivery system	**Lentiviral / Retroviral**	Stable long-term expression, efficient in dividing/non-dividing cells (80, 81).	Insertional mutagenesis risk, payload size limits (82, 83).	Durable cytokine/chemokine overexpression (e.g., IL-37) (79, 84).
	**Adenoviral**	Large payload, high efficiency (85, 86).	Transient expression, immunogenicity (87).	Short-term functional boosts or priming (85, 86).
	**Plasmid**	Low immunogenicity, suitable for RNP (88).	Lower efficiency, transient effect (89).	Transient modulation without integration (88).

CRISPR/Cas9 (Clustered Regularly Interspaced Short Palindromic Repeats/CRISPR-associated protein 9), TALENs (Transcription Activator-Like Effector Nucleases), and ZFNs (Zinc Finger Nucleases) are outlined with their advantages, limitations such as off-target edits or double-strand break (DSB) toxicity, and contexts of optimal use. Delivery strategies include lentiviral and retroviral vectors, adenoviral vectors, and plasmid-based ribonucleoproteins (RNPs). Additional considerations such as AI (Artificial Intelligence)-guided guide RNA (gRNA) design and immunogenicity risks are noted. References correspond to supporting evidence for each approach.

### Opportunities for MSC CRISPR/Cas9 modification in SLE therapy

As indicated in **Table 5**, recent applications of CRISPR/Cas9 across diverse stem cell types highlight the effectiveness of genetic modification in probing developmental mechanisms and modelling disease. For example, targeted knockouts in mammary stem cells and ESCs have clarified pathways regulating tissue differentiation and congenital disorders, while precise mutations in hematopoietic stem cells and iPSCs have revealed therapeutic avenues for blood, neurological, dermatological, and cardiac conditions. Collectively, these studies demonstrate that CRISPR/Cas9 enables both functional dissection of complex biology and the development of translational strategies. Building on this success, similar approaches could be applied to MSCs, where precise gene modification may enhance immunoregulatory properties and overcome current barriers in treating SLE.

**Table 5:** Modulation and methodology of CRISPR/Cas9 modification of stem cells.

Genes modified	Stem cell type	Modulation and method of CRISPR/Cas9	Findings	References
SOX2, PAX6, OTX2, AGO2	Human iPSC, ESC	Knockout, electroporation	Multiple genes can be targeted for inducible knockout Inducible gene knockout can occur in all cells at any differentiation stage	(102)
IDO	Human MSCs	Knockout, plasmid transfection	MSCs alter immune regulatory function against RSV by IDO MSCs affect immune cell proliferation	(103)
Ptpn22, MII3	Mammary stem cell organoid	Knockout, doxycycline-inducible lentiviral vector	Disruption of mammary gland differentiation Increased stem cell activity Activating the HIF pathway	(104)
HES1, ARX, GLIS3, MNX1, NGN3, PDX1, RFX6, PTF1A	Human ESC	Knockout, Doxycycline inducible Cas9	Supports systematic genome editing application for the understanding of mechanisms underlying congenital disorders PNDM and RFX6 affected pancreatic progenitors’ formation, and their further differentiation into functional endocrine cells Haplo-insufficient requirement for PDX1 in pancreatic endocrine differentiation	(105)
GFI1B	Hematopoietic stem cells	Point mutation	Increased megakaryocyte differentiation and platelet production	(106)
PTPS, DHPR	Human iPSC	Point mutation, electroporated Cas9 expression vector	Decreased tyrosine hydroxylase protein and extracellular dopamine levels	(107)
COL7A1	Human iPSC	Frameshift mutation, electroporated plasmid	Feasible in the development of autologous therapies for RDEB Efficient differentiation of iPSCs to somatic cells by CRISPR/Cas9	(108)
SLCO1A2, SLCO1B3	Human iPSC	Knockdown, Lentivirus expressing Cas9-sgRNA	Uncovered and validated a role for cell surface transporters SLCO1A2 and SLCO1B3 in doxorubicin-induced cardiotoxicity Decreased cell death in iPSC-derived cardiomyocytes	(109)

iPSC, induced pluripotent stem cells; sgRNA, single-guide ribonucleic acid; RDEB, Recessive dystrophic epidermolysis bullosa; ESC, embryonic stem cells; HIF, hypoxia-inducible factor; RSV, respiratory syncytial virus; IDO, indoleamine-2,3-dioxygenase.

CRISPR/Cas9 enables precise and versatile gene editing across multiple stem cell types. Applications span from understanding disease mechanisms to enhancing therapeutic functions, illustrating its translational potential for MSC-based therapies.

### Risks and limitations of CRISPR/Cas9

Although CRISPR/Cas9 is widely used for gene editing due to its effectiveness, it carries certain risks. One of the major concerns is off-target effects, which can disrupt the function of essential genes, leading to unintended consequences. Additionally, incomplete editing remains an issue, resulting in partial modifications that can compromise the therapeutic potential of the treatment and cause unpredictable outcomes. When CRISPR/Cas9 induces a double-strand break (DSB) in the DNA, the repair process can lead to insertions or deletions, potentially increasing the risk of oncogenesis. Moreover, the introduction of the Cas9 protein and guide RNA can provoke an immune response in the host organism. These risks associated with gene-editing tools are concerning, highlighting the need for improved strategies to mitigate these challenges. Despite advances, existing MSC-based therapies still face limitations such as heterogeneity, lack of standardisation, and variable clinical outcomes. To address these challenges, we have conducted a focused narrative review to synthesise advances in artificial intelligence (AI) to improve MSC modification for potential therapeutic use in SLE. Literature was identified through PubMed, Scopus and Web of Science searches using a combination of the terms “artificial intelligence”, “machine learning”, “deep learning”, “neural network”, “predictive modelling”, “mesenchymal stem cells”, “systemic lupus erythematosus”,” lupus”, and” cell therapy”. Only English-language articles were included.

## Artificial intelligence

Artificial Intelligence (AI) has emerged as a transformative tool in biomedical research, revolutionising how problems are approached and solved. AI refers to systems capable of performing a wide variety of tasks typically done by humans, simulating human cognitive abilities while operating autonomously (110). AI technologies include machine learning, natural language processing, and deep learning, which enable the system to learn from existing data, make decisions, and improve outputs over time without explicit programming (111)At the core of AI is machine learning, which involves analysing large datasets, recognising patterns, and producing predictions and decisions. Machine learning is further categorised into supervised and unsupervised learning. Supervised learning involves training algorithms on labelled data, while unsupervised learning detects hidden patterns in data without specific instructions or human involvement (112). Another branch, reinforcement learning, enables AI systems to learn through trial and error, generating actions based on feedback (113, 114). Deep learning, a subset of machine learning, uses artificial neural networks with multiple layers to model complex, non-linear relationships in data (115). Neural networks mimic the human brain, with layers of “neurons” processing data inputs (116). Deep neural networks (DNNs) are particularly powerful, as they employ many hidden layers to learn hierarchical representations of data. Initial layers learn basic features, while subsequent layers combine them to identify more abstract concepts, enabling deep learning models to achieve unprecedented accuracy (116). Deep learning is renowned for handling vast, complex datasets and detecting patterns across millions of data points without manual feature engineering (117, 118). One of its key advantages is its ability to continuously learn and evolve with the input of more data. The system can automatically adjust and improve based on new inputs, making it highly adaptable and effective over time (119).

## Role of AI in SLE treatment

SLE is a complex autoimmune disorder with heterogeneous symptoms and manifestations that affect multiple organs (3). AI can help in understanding and managing the complexity of the disease by analysing a large group of multi-modal datasets, including clinical, genetic and biomarker information, to identify patterns and correlations (120). The heterogeneous nature of SLE means the treatment options vary among patients (121). AI can significantly tailor personalised treatment plans based on individual patient profiles, leveraging diverse datasets for precise treatment selection, improving efficacy and reducing the risk of adverse effects (122). Several studies have demonstrated the potential of AI in the diagnosis and treatment of SLE (**Table 6**).

**Table 6:** Applications of machine learning models in systemic lupus erythematosus (SLE). Different ML models in SLE research are summarised, listing down their input, output and key findings.

ML model	Study / application	Input data (independent variables)	Output (dependent variable)	Key findings / uses
Random Forest (RF)	SLE risk prediction	Genetic markers, family history, environmental factors, clinical features	SLE development	Identifies individuals at risk of SLE development (90)
	LN prognosis	Age, treatment type, duration, lab results, treatment histories	LN outcomes (renal function progression)	Predicts LN outcomes (91)
	Flare prediction	Clinical/lab data	Risk of lupus and renal flare	Predicts lupus and renal flares (7, 92, 93)
Gradient Boosting Machines (XGBoost)	LN 1-year outcome prediction	Demographics, treatment type, lab results, EHR data, clinical metrics	1-year LN outcome (positive/negative)	Improves prediction by sequential boosting (94)
LASSO Regression	Renal flare risk stratification	Biomarkers, age, treatment type, patient history	Renal flare risk	Prevents overfitting, selects significant predictors (95)
Artificial Neural Networks (ANNs)	Allograft survival	Demographics, history of transplant, treatment response	3-year allograft survival	Predicts kidney transplant outcomes in SLE patients (96)
	Hospital readmission	EHR data (treatment history, demographics, disease severity, comorbidities)	Hospital readmissions	Predicts hospital readmissions and captures long-term dependencies in sequential data (97)
Long Short-Term Memory (LSTM)	Hospital readmission (time-series)	Time-series EHR data (treatment history, outcomes)	Hospital readmission	Captures long-term dependencies in sequential data (97)
K-means Clustering	Disease activity grouping	Genetic markers, biomarkers, clinical signs	Risk of disease activity	Groups patients by disease activity risk (98)
Sparse PLSDA	Disease activity classification	Clinical data, biomarkers, patient history	High *vs* low disease activity	Classifies patients by disease activity (99)
Recurrent Neural Networks (RNNs)	Chronic damage prediction	Longitudinal clinical data, lab results, patient history	Chronic damage progression	Predicts long-term chronic damage in SLE (100)
Support Vector Machines (SVMs)	Disease control factors	Demographics, disease severity, treatment regimens	Comprehensive disease control	Identifies factors influencing disease control (101)

Several studies have used Random Forest, which is an AI model that constructs multiple decision trees to improve prediction accuracy and handle high-dimensional data (123). Studies use this predictive model to identify individuals at risk of SLE development using genetic data and clinical features, environmental factors and family history that are fed into the algorithm. The data includes the dependent variable, which is SLE development, along with independent variables such as genetic markers, family history, and environmental factors (90). RF has also been used in predicting outcomes of lupus nephritis (LN) based on clinical variables, treatment histories, and lab results, with dependent variables being lupus nephritis outcomes (renal function progression) and independent variables such as age, treatment type, duration, and lab results (91). It also predicts the early flare of lupus (124) and stratifies the risk of renal flare (95). Apart from RF, the use of Gradient Boosting Machines (XGBoost) was recorded in studies predicting the 1-year outcomes of lupus nephritis (LN) based on EHR data, clinical metrics, laboratory data, and treatment data. The dependent variable is based on the 1-year lupus nephritis outcome (positive/negative), while the independent variable is the demographic data, treatment type, and lab results. XGBoost is used to boost algorithms that build models sequentially to improve accuracy by correcting prior errors (94). Although powerful, tree-based methods may be difficult to interpret, sensitive to class imbalance and at risk of overfitting if not carefully tuned (125, 126).

To overcome the risk of overfitting, LASSO regression applies a penalty to reduce overfitting and retain only the most significant prediction. In SLE, it has been used to stratify renal flare risk using biomarkers, clinical variables, and patient history as the dependent variable while biomarker, age and treatment type as the independent variable (95). While these models are transparent and effective in small datasets with many variables, they may discard relevant but weaker predictors that can be unstable across validation folds (127). Studies have also shown the use of artificial neural networks (ANNs) that mimic brain-like processing with layers of neurons to forecast outcomes based on patterns in data. Several studies have been recorded using Artificial Neural Networks (ANNs) successfully.

A study by Tang, Poynton (96) predicted the survival rate of a 3-year allograft in kidney transplant recipients. The predictive modelling used data mining methods, such as classification trees, logistic regression, and artificial neural networks, to analyse the data of recipients with SLE and kidney-related complications. The data used for ANNs is patient demographics, clinical parameters, and transplant history, with allograft survival (3 years) being the dependent variable and demographics, history of transplant, and treatment response acting as independent variables (96). ANNs were also used in predicting hospital readmissions of SLE patients. ANNs processed EHR data that includes patient history, treatment, and clinical outcomes, with hospital readmissions as the dependent variable and treatment history, demographics, disease severity, and comorbidities as independent variables (97). Long Short-Term Memory (LSTM) was also used to predict hospital readmission by retaining long-term dependencies in data that are critical for time-series predictions (97). Despite their significant value, deep learning models require large, well-curated datasets and are prone to overfitting in small study groups (128).

K-means Cluster Analysis is another significant AI model that uses clustering techniques to group similar cases for pattern identification. A study by Toro-Dominguez, Martorell-Marugan (98) identified the risk of disease activity using data obtained from genetic data, clinical records, and biomarkers. The key dependent variables are the level of disease activity risk, while the independent variables are genetic markers, biomarkers, and clinical signs that are fed into the AI model. One of the limitations is that it requires longitudinal gene expression from multiple time points per patient, limiting its clinical applicability to classify new patients (98). Similar studies were done using Sparse Partial Least Squares Discriminant Analysis (PLSDA),which combines dimensionality reduction with discriminant analysis to classify high disease activity based on clinical and biomarker data and patient history (99).

Apart from that, Recurrent Neural Networks (RNNs) were used to predict chronic damage in SLE. RNNs use feedback loops to process sequential data and predict long-term chronic damage. The data that was used for the AI model includes longitudinal clinical data, lab tests, and patient history. The dependent variable is the progression of chronic damage over time, while the independent variables are the disease activity, lab results, and treatment history (100). Other AI models involved in SLE prognosis include support vector machines (SVM) that were used to identify relevant factors influencing lupus that provide a comprehensive disease control achievement. The data include patient demographics, clinical outcomes, and treatment regimens, based on dependent variables (comprehensive disease control) and independent variables (age, severity of disease, and treatment regimens). SVM finds the optimal hyperplane to classify data and identify key factors influencing disease control (101). SVMs are usually effective in medium-sized datasets. SVMs are sensitive to parameter selection, class imbalance and are generally difficult to interpret beyond linear models (129). Below, we present **Table 5**, simplifying the above corpus for easy readability.

The success of these AI models in predicting SLE prognosis and treatment highly depends on robust data pre-processing techniques, given the complex, high-dimensional and multi-modal nature of SLE datasets. Data cleaning is an important step in data pre-processing as it removes or corrects errors, missing values and inconsistencies, ensuring the integrity of the datasets. For instance, certain clinical data may contain missing patient records and inconsistent lab results that need to the addressed. Thus, imputation methods are used to fill in missing values, especially in large datasets that are incomplete. Common imputation methods include median imputation, data removal and multiple imputation using chained equations (130). Moreover, data normalisation is done in AI models, particularly RF or SVM, to prevent bias by variables with larger numerical ranges. Data normalisation ensures variables measured on different scales are transformed into a consistent range (131). Apart from that, machine learning utilises feature selection, which plays a significant role in reducing dimensionality, allowing AI models to focus on the most relevant variables. This approach enables AI models to improve performance by concentrating on the most relevant variables. Common methods include feature elimination (RFE) and principal component analysis (PCA), which help in reducing noise and highlighting key predictors of SLE progression or treatment response (132). Data augmentation is also used to enhance model generalisation by artificially increasing the datasets through techniques like bootstrapping or introducing slight variations in data, especially in small sample sizes in SLE datasets. Splitting the data into training, validation and test sets can ensure unbiased AI model performance and generalise well in new data (132). These data pre-processing steps can help AI models effectively handle SLE’s complexity.

The advances in AI applied to SLE integrate complex datasets ranging from genetic and biomarker profiles to predicting disease risk, stratifying patients and forecasting outcomes. Importantly, many of these approaches converge on immune pathway activation and patient heterogeneity, which are also a central part of the response to cell-based therapies. The ability of AI to uncover mechanistic insights into patient-specific patterns provides a foundation for extending its use in MSC therapy, where optimising immunomodulatory function and tailoring interventions to individual disease states remain key challenges.

## Role of AI in MSC modification

**Figure 1:**
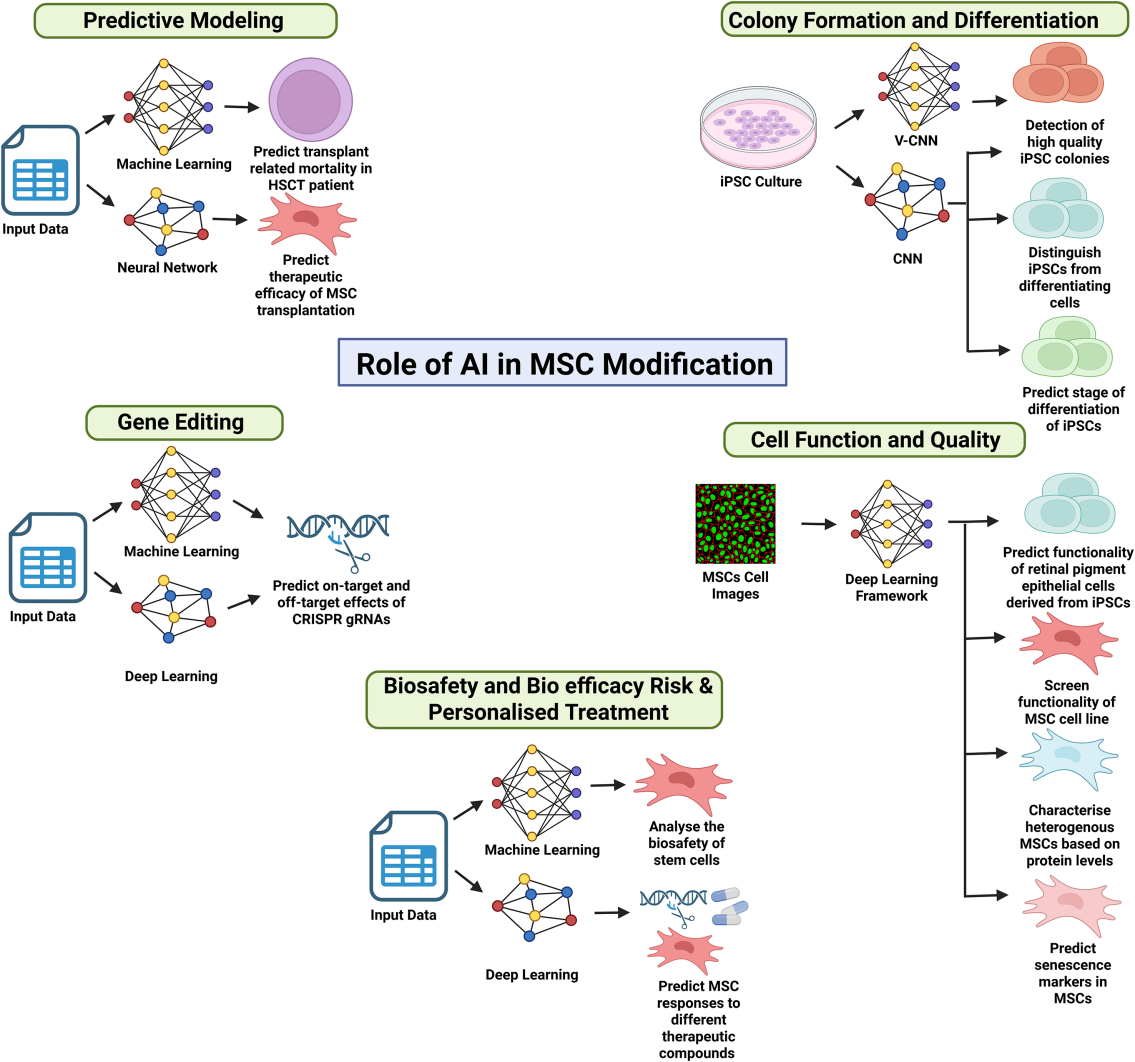
Schematic diagram of the studies on the role of AI in MSC Modification. The schematic figure summarises different roles of AI in the modifications of MSCs, such as gene editing, cell function and quality, predictive modelling and colony formation and differentiation. (Created using “Biorender.com”).

AI is revolutionising the field of MSCs modification by providing innovative solutions to enhance the precision, efficiency, and scalability of genetic and cellular alterations. MSCs are widely studied for their potential in regenerative medicine and autoimmune disease therapies due to their immunomodulatory and differentiation capabilities. However, the optimisation of MSCs for specific therapeutic outcomes presents numerous challenges, including off-target effects in gene editing, predicting cell behaviour, and personalising treatments (78, 133). AI, particularly through machine learning (ML) and deep learning (DL), is increasingly being applied to address these challenges, offering powerful tools for accelerating MSC modification and improving therapeutic outcomes that can be observed in **Figure 1**. A summary of ML and DL tools used in MSC modification can be observed in **Table 7**.

**Table 7:** ML and DL applications in fine-tuning MSC modification.

Task	ML/DL model	Input data	Output	Key findings
Predict 100-day mortality after HSCT	Alternating Decision Tree (ADT)	Clinical variables: disease stage, donor type, CMV serostatus, performance score	100-day mortality	Outperformed EBMT score; interpretable predictions; personalised risk estimation (126)
Predict MSC transplantation efficacy in cartilage repair	Neural Network	Cell number, defect area, defect depth, patient body weight	Therapeutic efficacy, optimal implantation dose	Imputed missing data, guided personalised dose recommendations (134)
Detect iPSC colony formation	CNN	Time-lapse culture images	Colony detection	Higher accuracy than traditional methods (135)
Detect high-quality iPSC colonies	V-CNN	Stem cell culture images	Colony quality classification	Accurately identified high-quality colonies (136)
Distinguish between pluripotent *vs* differentiating stem cells	CNN	Microscopy images	Differentiation stage	99% classification accuracy (137)
Predict iPSC-to-hepatocyte differentiation stage	CNN	Morphological features	Differentiation stage	Enabled real-time monitoring of differentiation (138).
Predict the function of RPE cells from iPSCs	Deep Neural Learning	Quantitative microscopy images	Functional quality	Rapid, non-invasive functionality prediction (139)
Screen MSC functionality	End-to-end DL	Live-cell microscopy	Functional classification	Ensured therapeutic quality before transplantation (140)
Convert microscopy images to protein-level data	DL analysis	Light microscopy images	Protein quantification	Enabled assessment of MSC heterogeneity (141)
Predict MSC senescence markers	DL	Phase-contrast microscopy → immunofluorescence prediction	Senescent *vs* non-senescent classification	Accurately monitored senescence in real time (142)
Classify senescence states	Cascade R-CNN	Multicellular microscopy images	Senescence state classification	Automated single-cell detection and senescence classification (143)
Predict on-/off-target effects in CRISPR	ML/DL predictive models	gRNA features, sequence data, experimental conditions	On-target *vs* off-target predictions	Improved CRISPR specificity, optimised gRNA/Cas9 design (133–135, 144)
Enhance immunomodulatory effects of MSCs	ML/DL predictive models, i.e. DEEP-CRISPR/CRISPR-ML	MSC genome	↑ Migration, survival, proliferation, ↓ T-cell differentiation	AI-guided CRISPR editing enhanced MSC therapeutic traits (136, 137)

### Predictive modelling

AI-driven predictive models have proven to be highly effective in predicting outcomes in stem cell therapies. The study by Shouval, Labopin (144) proved the use of alternating decision tree (ADT) machine learning algorithms to a large cohort of patients undergoing allogeneic HSCT. The ADT model integrated clinical variables such as disease stage, donor type, cytomegalovirus serostatus and performance scores to predict 100-day overall mortality with higher accuracy than conventional EBMT scoring. The algorithm was not only predictive but also interpretable, enabling individualised probabilistic risk estimates through a user-friendly online interface. A similar predictive system has been developed for MSC therapies. For example, a neural network model was developed to predict the therapeutic efficacy of MSC transplantation in cartilage repair based on existing results in animal and human clinical trials. The model incorporated key factors such as implantation cell number, defect area, defect depth and patient body weight and was able to impute missing data while estimating prediction. Importantly, it provided clinicians with individualised predictions of therapeutic outcomes and recommended treatment parameters such as optimal implantation dose for effective repair. This AI-based model demonstrates how predictive modelling can guide personalised MSC therapies, assist in clinical decision-making and be adapted for broader applications (134).

### Colony formation and differentiation

Apart from predictive modelling, AI has been used to automatically detect induced pluripotent stem cell (iPSC) colony formation and differentiation by monitoring stem cell cultures more accurately. A study by Fan, Zhang (135) developed a non-invasive machine-learning model using CNN as a classifier to detect iPSC colonies more accurately than traditional methods. while Kavitha, Kurita (136) also developed a V-CNN machine-learning model that accurately detected high-quality iPSC colonies. Deep learning neural networks have also proven to be highly accurate in identifying early stem cell differentiation. A study by Waisman, La Greca (137) used convolutional neural networks (CNNs) to distinguish pluripotent cells from differentiating cells with 99% accuracy. These studies demonstrate how AI integrates data acquisition (time-lapse imaging of culture), modelling (CNN-based classification), and feedback (real-time colony quality assessment) to support cell culture decision making. Translating this workflow to MSC cultures, AI could enable real-time monitoring of cell state transitions, ensuring cells retain their therapeutic phenotype and inform time interventions such as media replacement, supplementation or reprogramming (145). A similar study used a similar deep learning model to predict the stage of differentiation of iPSCs that were undergoing differentiation towards hepatocytes based on morphological features of cell cultures (138).

### Cell function and quality

Apart from early detection of cellular differentiation, AI can be adapted with non-invasive techniques to predict cell function and quality during MSC-based therapies. MSCs are known for their heterogeneity in functions and lack appropriate standardisation methods of MSC lines. A study by Schaub, Hotaling (139) demonstrated that deep learning models could predict the function of retinal pigment epithelial cells derived from iPSCs using quantitative microscopy by analysing cell images to assess MSC functionality, providing a rapid and reliable method to ensure the therapeutic quality of cells before transplantation. A similar study developed an end-to-end DL framework to screen the functionality of MSC cell lines based on images obtained from a live-cell microscope (140). Another study recorded the use of AI to convert transmitted light microscopy images of the protein levels of MSCs into measurements that can be quantified to characterise the heterogeneous MSCs (141). The heterogeneity of MSCs also affects the rate of senescence of MSCs. A study by Weber, Lee (142) developed an AI model that predicts the immunofluorescence images of senescence markers in MSCs that are obtained from phase contrast images. The AI model successfully differentiated the senescent and non-senescent population of MSCs, which can further improve the therapeutic potential of MSCs. Complementing this, a separate study introduced a morphology-based Cascade R-CNN algorithm that automatically detects single cells of varying shapes within multicellular images and classifies their senescence state (143). These methods illustrate how image-based deep learning can be integrated into the MSC processing pipeline for real-time senescence monitoring.

### Gene editing

Gene editing is a powerful tool that allows precise insertion, removal and deletion of genes in a DNA sequence (92). The most advanced gene-editing technologies include the zinc-finger nucleases (ZFNs), transcription activator-like effector nucleases (TALENs), and CRISPR-Cas-associated nucleases (CRISPR/Cas9) (93). Among these 3 gene-editing technologies, CRISPR/Cas9 is the most used gene-editing technology that is easy to use and more effective comparatively (76). CRISPR technology has shown an upward trend and positive progress, producing numerous clinical trials (77). Gene editing can be used to treat multiple human diseases, especially those caused by genetic mutations. CRISPR can knock down defective genes and replace these genes in the cells with new genes in diseases such as sickle cell anaemia and thalassemia. Furthermore, CRISPR can target genes responsible for metabolic syndrome, neurodegenerative diseases and cancer. Lastly, the successful use of CRISPR has been recorded in treating immune system diseases such as acquired immunodeficiency syndrome AIDS caused by human immunodeficiency virus (HIV) by creating resistant cells to infections (77, 146). The notable outcomes of CRISPR/Cas9 in other diseases have paved the way for its potential use in SLE. A study by Harris, Koelsch (147) used CRISPR-Cas9 to knock down CXorf21 genes that are involved in the X chromosome in SLE disease. The knockdown of this gene led to the reduction of TNF-α and IL-6 expression.

Although gene editing technologies have shown potential for future treatment, the risks of these technologies are very much apparent. The risk of off-target effects is high due to the potential of Cas9 to bind and cleave unintended genomic binding sites that lead to undesirable gene functions (148). To overcome this risk, AI can be integrated into the CRISPR/Cas9 to regulate and refine numerous gRNA features that are identified to affect the binding and cleaving efficiency of gRNA that produces off-target effects. AI using predictive algorithms can predict various on-target and off-target effects of CRISPR gRNAs in silico thus, improving its specificity (149). The gRNA is highly affected by many factors, such as cellular environment, gRNA, sequence of target and experimental condition (150). Machine learning models can include all these data from various factors to successfully predict the on-target and off-target effects. Machine and deep learning-based algorithms have been successfully used in CRISPR technologies to predict on-target efficacy (47, 150).

Optimising gene editing technologies using AI can significantly improve its potential to be developed as personalised medicine. AI can predict treatment responses of patients depending on their genetic profiles and health history. CRISPR can modify genes depending on the profile of individual genes (151). The notable potential of integrating AI in CRISPR poses benefits in optimising gRNA designs, Cas9 variant selection and the prediction of potential off-target sequences (152).

The integration of AI and CRISPR addresses the limitations of CRISPR technology while creating a safer alternative in the world of genome editing. The potential of gene editing technologies has paved the way for improving MSCs by gene modifications. CRISPR can improve MSCs by editing certain genes to express enhanced immunomodulatory effects via the effect of IFN-r priming (153). Apart from enhancing the immunomodulatory effect, CRISPR/Cas9 can manipulate genes that are related to migration, survival, proliferation and triggering either anti-inflammatory or pro-inflammatory responses while reducing the differentiation of T cells (153, 154). These findings suggest the possible improvement of CRISPR editing of MSCs using AI to ensure desired outcomes in MSC modifications.

### Biosafety and bio efficacy risk & personalised treatment

The use of MSC therapies poses serious biosafety risks due to their risk of differentiating aberrantly and the potential adaptation to the microenvironment, possibly aggravating any existing condition. AI has the potential to assess the biosafety and bio-efficacy of MSC therapies, ensuring that modifications made to MSCs do not lead to unintended side effects such as tumorigenesis and teratogenesis (47).

### AI-guided MSC engineering- current efforts and potential improvements

Incorporating AI into MSC research can help identify optimal modification techniques while optimising the microenvironment for the development of stem cells without compromising cellular integrity. AI also plays a role in drug screening and treatment personalisation. AI models ‘predict’ via noticing patterns, therefore multi-omic datasets (transcriptomic, proteomic and epigenetic profiles) can be added for AI to form meaningful analyses, uncovering subtle determinants of MSC fate and functionality that are obscure through conventional means (155). This integration could potentially serve as regulatory checkpoints that dictate MSC differentiation, proliferation and immunomodulatory potential, which can inform the design of targeted interventions for disease-specific applications.

AI can also predict MSC responses to different therapeutic compounds and gene editing techniques (156). For example, applied machine learning has analysed the effects of drugs on iPSC-derived cardiomyocytes, which achieved a classification accuracy of 79%. This model can be adapted to MSC-based therapies to predict the responses of cells to various drugs or gene-editing techniques (157). Similar approaches could in theory be adapted following the 4 classifications above, reducing time and effort taken on *in vitro* and *in vivo* testing.

Beyond drug response prediction, AI has also been applied to modelling immune pathway activation, which might hold promise in informing MSC engineering. Previous efforts been successfully done in the profiling of sepsis immunity using supervised learning algorithms such as Gradient Boosting Trees (158). Another proof of concept was done by Yifeng Tang et al., whose group successfully applied quantitative structure–activity relationship (QSAR) models to identify innate immunomodulators. Signalling cascades that are most relevant for enhancing or suppressing specific immune responses can be identified, which would largely guide the rational design of MSC modifications. Pathways that serve as negative feedback loops for aberrant inflammation for example can be upregulated, which would serve fastidiously in stamping out the problem.

The advent of machine learning models can pioneer the personalisation of MSC-based therapies, tailoring treatment regimens to the individual’s cellular microenvironment and genetic profiling for better outcomes. These noteworthy outcomes of AI in MSC modification can be potentially applied to further improve MSC treatment in SLE. Tailoring treatments could potentially be done at a holistic and in-detail manner, maximising efficacy while minimising risks such as flare-ups or tumorigenesis. This invokes a paradigm shift toward precision cellular medicine, which holds great potential in the transformation of MSC therapies from experimental interventions into safe, standardised and patient-centric treatments.

## The future potential of AI-guided modification of MSC in the therapy of SLE

The modification of MSCs has emerged as a promising approach in regenerative medicine, yet the outcomes of these modifications vary significantly across different methods. This inconsistency is largely due to the intricate interplay between MSCs and the imposed modifications, which can alter the autocrine and paracrine signalling mechanisms within the culture. Traditionally, researchers have relied on fundamental principles of immunology and molecular biology to guide these modifications, often making educated guesses that, while effective in a general context, still leave considerable room for optimisation.

**Figure 2:**
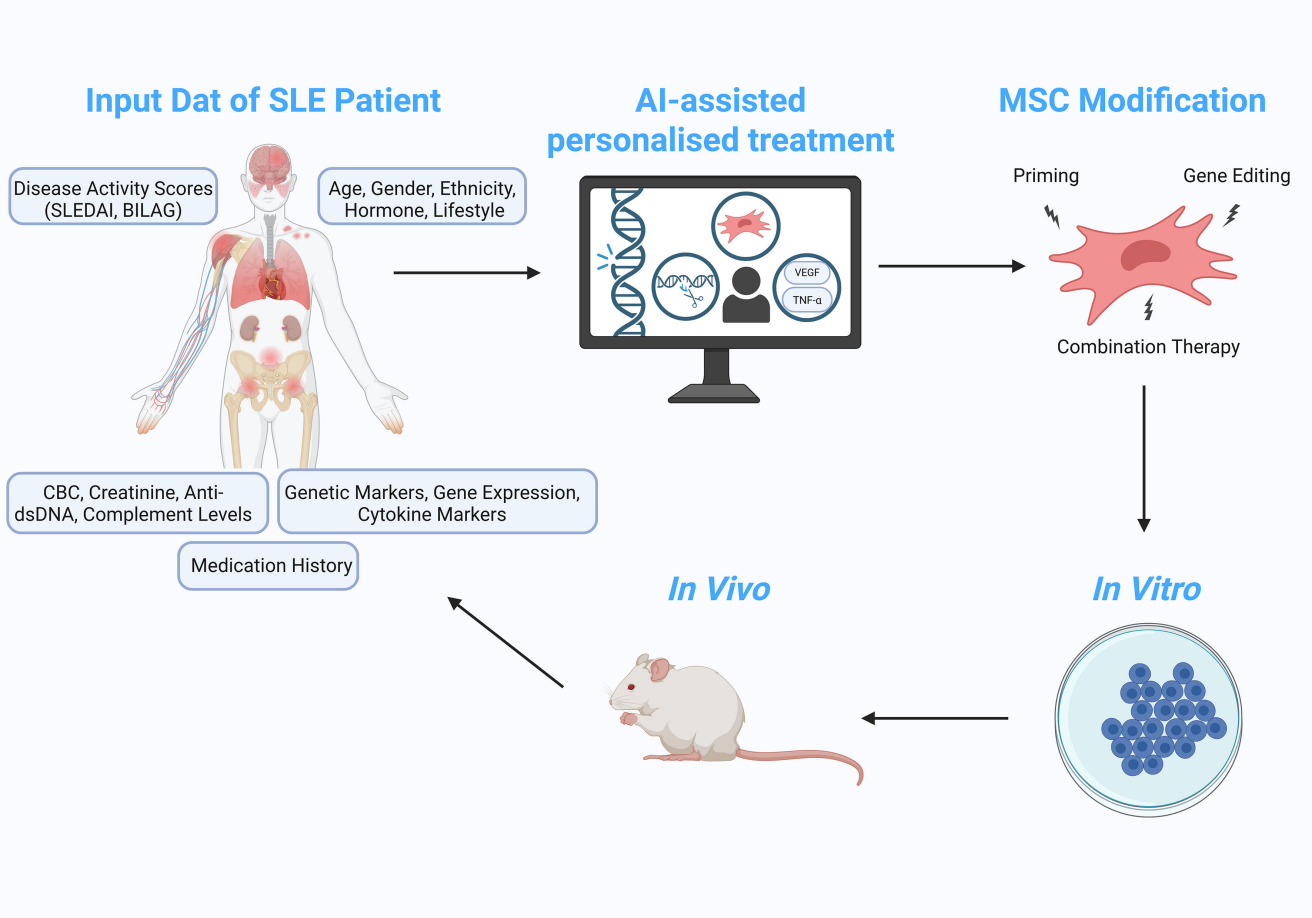
Visual Representation of Conceptual Idea Involving the Use of AI in Improving MSCs Modifications. The idea includes deriving input data such as disease activity score, age, gender, ethnicity, hormone, lifestyles, genetic markers, gene expression and cytokine markers, as well as CBS, creatinine, anti-dsDNA and complement levels that will be analysed using an AI model. The MSC modification is tailored accordingly based on the specific data of the patient that was obtained for personalised medicine. (Created using “BioRender.com”).

This is where AI, particularly deep learning, offers exciting potential. By analysing vast datasets of past successes and failures, AI can provide data-driven predictions or even perform zero-shot learning, inferring optimal modification strategies for MSCs. As AI systems continuously improve, they can suggest increasingly effective approaches tailored to specific conditions, such as SLE. Over time, these models will become more refined, offering more precise and cost-effective solutions, while also reducing the time and resources needed to develop individualized treatment plans. This integration of AI holds the promise of revolutionizing MSC modification strategies for the treatment of SLE, driving both deeper scientific understanding and clinical breakthroughs (**Figure 2**).

## Identifying key pathways and gene modules for targeting

Machine learning has significantly proven to predict SLE disease activity based on gene expression data to overcome the issue of heterogeneity among patients. AI models like generalised linear models (GLM), k-nearest neighbours (KNN) and RF classifiers were trained on SLE patient gene expression data from multiple SLE datasets to identify important genes involved in SLE disease activity. These gene expression profiles must be understood as they provide a potential for therapeutics in SLE (159). Critical genes associated with SLE activity that are identified through machine learning can serve as targets for gene editing technologies. For example, a study used BioGPS, STRING database, Protein-Protein Interaction (PPI) and KEGG enrichment analysis to identify potential therapeutic genes for the treatment of SLE. MSCs can be modified specifically to carry these therapeutic genes that can be expressed, increasing their efficacy in treating SLE (160). Moreover, overexpressing certain enzymes in MSC-derived extracellular vesicles could enhance their therapeutic potential for SLE patients (161). miRNAs in MSC-derived extracellular vesicles (EVs) could be an effective tool for both the identification of key pathways and gene modules that mediate the pathogenesis of SLE. AI can be used to understand the different miRNAs, such as miR-146a-5p, miR-19b, and miR-20a, that are required to be overexpressed or inhibited in MSC-derived EVs for improved therapeutic potential in SLE patients (162, 163). The use of AI to identify these miRNAs could revolutionise the discovery of more therapeutic miRNAs and understand their effects and outcomes in SLE patients before treatment.

Understanding gene expression profiles identified by machine learning of SLE patients potentially allows for the priming of MSCs to enhance their therapeutic efficacy. By upregulating genes involved in immune modulation, MSCs can be tailored to better meet the therapeutic needs of SLE patients. For example, priming MSCs with IFN-γ has been shown to increase the expression of class II HLA molecules, contributing to immune system homeostasis (164). Moreover, machine learning classifiers can assess the efficacy of primed MSCs by analysing their post-priming gene expression profiles. By comparing these profiles to established markers of MSC efficacy, machine learning models can predict the potential of primed MSCs to modulate SLE activity effectively.

## Improving MSC therapy precision through predictive models

A Swedish study (165) obtained genotype data from SLE patients (1160 people) and healthy controls (2711 people) using the Illumina Immunochip. After quality control, 134523 SNPs that are either located in or are close to 125000 genes related to the immune system are selected for further analysis. The random forest model is chosen to first classify individuals as either SLE patients or healthy individuals, with its performance evaluated using the Area Under Curve metric (AUC), which ranges from 0 to 1, with 0.5 indicating a random prediction and 1 representing a perfect prediction. The model scored 0.78 in SLE prediction, which was better than the logistic model (p-value 0.0028, DeLong’s test), which only scored 0.74. AUC also reached 0.91 for the random forest model in the detection of nephritis within SLE patients, compared to the logistics model which only scored 0.70. The ability of random forest models to predict SLE through AUC suggests that predictive biomarkers derived from genotype or gene expression data are accurate and feasible. This information could lead to patient stratification based on genetic and gene expression profiles that could optimise MSC therapy, tailoring treatment to those most likely to benefit. The predictive biomarkers can potentially create a biomarker-guided selection of MSC (e.g., based on secretome profiles or expression of specific immunomodulatory genes) that can enhance therapeutic efficacy.

After successful prediction, the study proceeded to use AI to identify risk genes for SLE by quantifying how strongly a gene region contributes to SLE risk based on SNP genotype data. 40 top genes associated with SLE were identified, and from within 25 known SLE-associated genes were validated, and novel candidate genes (i.e. ZNF804A, ANK3 and MANF) were identified. 12 of the top 40 genes were associated with other autoimmune diseases.

Within the 40 genes, 15 have differential expression between B cells and T cells (enrichment in B cells), and 30 were expressed in B or T cells. 6 were regulated by cis-regulatory SNPs. These risk genes showed enrichment for allele-specific expression and cell-type-specific regulation, supporting functional relevance in SLE pathogenesis. This study proved the potential of AI in identifying differentially expressed genes in B and T cells, and cis-regulatory SNPs linked to immune cell-specific activity in SLE. This information could potentially lead to the engineering of MSCs to express factors that specifically modulate B and T cell activity and target cytokine signalling pathways that are identified as critical in SLE pathogenesis.

## Targeting immune cell populations

In another study by Kegerreis, Catalina (159), SLE disease activity is predicted using gene expression data from Gene Expression Omnibus (GEO), taking into consideration active and inactive SLE (SLEDAI ≥ 6 for active,<6 for inactive). The data selected consists of purified cell populations (CD4, CD14, CD19, CD33 and LDG) and whole blood (WB) samples. R statistical package is used for Quality Control, normalisation and filtering on the raw microarray data, and then LIMMA R packaged is used to perform Differential Expression analysis (DE). Weighted Gene Co-expression Network Analysis (WGCNA) is then used to identify gene modules in purified cell populations. Gene Set Variation Analysis was then conducted to test the enrichment of cell-specific gene modules in WB datasets. 3 classifiers are trained and validated through 10-fold cross-validation and study-based cross-validation, with them bringing Elastic Generalised Linear Model, k-Nearest Neighbours and Random Forest.

The clustering of patients based on DE genes fails to reliably separate active from inactive disease states, and although several gene modules correlate with SLEDAI, they cannot fully distinguish active and inactive patients in individual analysis. Random Forest achieved 83% accuracy using raw gene expression data and identified critical genes and modules to SLE pathogenesis, which include interferon-related pathways and monocyte-derived modules, both positively and negatively associated. The use of AI to identify the gene modules associated with the leukocyte population monocyte-derived modules, interferon-related pathways) could assist in the engineering of MSCs to target immune cell populations. For instance, MScs could be modified to reduce the activity of monocytes, which play a significant role in autoimmune response in SLE. MSCs could also be modified to regulate B and T cells that reduce autoantibody production and T cell activation. Thus, improving patients’ clinical outcomes.

The noteworthy evidence of the use of AI in SLE represents a significant advancement, as it enables the analysis of large and complex datasets to identify critical insights. These insights can be leveraged to modify MSCs, tailoring their therapeutic properties to align with the specific needs of individual patients. By integrating Al-driven data analysis with MSC therapy, a more personalised and effective treatment approach for SLE can be achieved, ensuring the therapy is optimised based on patient-specific genetic and molecular profiles identified through AI.

## Challenges and ethical considerations

The use of gene-editing approaches, which although powerful, remain imperfect. Off-target editing or insertional mutagenesis have yet to be completely resolved. For mitigation, orthogonal detection platforms, such as GUIDE-seq and CIRCLE-seq should be utilised, accompanied by stringent release criteria as suggested by FDA and EMA as part of Advanced Therapy Medicinal Products oversight, with the related guidelines cited here (166–168). A lot of the clinical trials also demonstrated donor-specific HLA antibodies following allogenic MSC infusion (author’s observation). This presents a problem that limits translation, immunogenicity. Although so far, no adverse events have been observed due to such immunogenicity, such phenomenon warrants caution. Care should be taken to follow the EMA guidelines for cell- based therapies, placing emphasis on standardised immuno-monitoring as well as using early-passage/hypoimmunogenic engineered MSCs to prevent adverse events.

Donor-related variability further increases the risk of such therapeutic option. As illustrated by heterogeneity in HPL expansion media, donor age and tissue source substantially influence MSC potency and reproducibility (169). Implementation of potency assays that are based on defined mechanisms of action, such as immunomodulation via IDO activity, or the expression of specific surface markers, are increasingly recognised as essential for lot release under ATMP guidance (170, 171). MSC procurement should also be done responsibly and ethically, covering all bases such as informed consent, fair compensation and transparent donor communication, to avoid repeating historical injustices such as the Henrietta Lacks case.

AI holds significant promise in enhancing the modification and personalisation of MSCs for SLE, yet its application is a double-edged sword. One significant challenge is the availability and quality of data. The functionality of AI highly depends on large, high-quality datasets, but, MSC research is often inconsistent, yielding incomplete data from different labs and clinical trials (author’s observation). The lack of quality data leads to the inaccuracy of AI models to make predictions and reliable conclusions regarding MSC behaviour, treatment outcomes and optimal conditions (172). It is important to ensure there is a large, open-source and standardised database to improve the quality of AI-driven insights (173). Furthermore, SLE is a highly heterogeneous disease with varying clinical representation based on patient genetics, ethnicity, sex, immune response and disease progress (174). AI models must account for this complexity to predict the modifications of MSCs in different patients. This limits the ability of AI to provide universally applicable solutions, thus increasing the difficulty in personalising MSC treatments for individual patients.

Apart from SLE, MSCs are highly heterogeneous with their behaviour influenced by numerous factors such as tissue origin, donor variability, culture conditions and disease environment (175–177). The complex biology of MSCs makes it difficult for AI models to predict MSC behaviour and their therapeutic effects accurately in diseases like SLE, where the immune system is a major component. The complexity of MSCs and SLE can be understood if AI models are developed to integrate multi-omics data (genomics, proteomics and metabolomics) to account for this complexity (178, 179). The integration of AI with biological systems is also a significant challenge. AI can predict optimal gene modifications, but validating these predictions in biological systems requires extensive experimentation due to the intricacies of biological validation. The FDA and MHRA have issued Good Machine Learning Practice (GMLP) principles that urge model calibration, external validation and transparent reporting.

Apart from challenges, the use of AI comes with the issue of patient data privacy. AI systems largely rely on datasets that include sensitive patient information such as genetic data and medical history (180). This information is considered confidential. Ensuring the confidentiality of this data while sharing it for AI model training poses significant ethical concerns (180). Strict laws must be developed to protect patient privacy and ensure compliance with regulatory requirements regarding data privacy (181). Frameworks such as the EU AI act and WHO guidance on AI ethics serve as good advice to secure data governance, federated learning and also strict regulatory compliance to maintain and safeguard confidentiality.

Furthermore, AI models can inherit biases from the data on which they are trained. If the training data predominantly represents certain demographics (ethnic or gender biases), AI may produce fewer effective treatments for underrepresented populations (182). This indirectly will worsen health disparities in SLE, which disproportionately affect women and certain ethnic groups (182). AI must be designed to reduce these biases to ensure fairness in MSC treatment. Moreover, it is of utmost importance that the patients are fully informed about the use of AI in developing their MSC-based treatment. Patients should understand the role of AI and its potential risks and uncertainties associated with AI decisions to maintain trust in AI-based medical innovations (183).

Accountability and liability are important ethical considerations for the use of AI in MSC modifications. It is important to establish accountability frameworks to manage risk associated with AI in medical research, especially in treatment involving modification of MSCs in complex diseases like SLE (184). The area of using AI to modify MSCs for the treatment of SLE is relatively new thus, long-term safety and ethical use of modified MSCs should be carefully considered. The long-term effects of genetically modified MSCs are still not fully understood. AI may optimise gene-editing techniques, but permanently altering cells for therapeutic purposes, especially in autoimmune diseases like SLE comes with ethical implications (185). Robust safety protocols must be established, and long-term monitoring must be done to ensure the modified MSCs do not cause tumorigenicity or immune dysregulation.

Finally, regulatory and real-world design considerations would determine whether AI-enhanced MSC therapy achieves safe translation. The updated International Council for Harmonisation E6 (R3) clinical trial guidance emphasises adaptive design, independent monitoring and harmonised data collection to support reproducibility across sites (186). EMA reflections on ATMP stress the importance of long term follow up to monitor tumorigenicity, immune dysregulation and other delayed effects (166). Vigilance should extend beyond conventional endpoints, encompassing algorithmic oversight to track when and how AI-driven recommendations deviate from expectations. Black-box and limited white-box access should also be given to regulatory bodies to further audit the data.

## Future directions and innovations

The application of AI to enhance MSC therapy for SLE offers promise to revolutionise both the development and clinical applications of MSC and the future of MSC, as well as their offshoot modifications to treat SLE. As mentioned above, AI can be a powerful tool to optimise MSC modification, culture, and application, thereby reducing costs, improving therapeutic efficacy, and personalising treatments to individual patients. A few directions are listed below for further reference.

### Combining different methods of MSC modification

SLE is a complex disease, being an intermeshing of various individual segregated conditions stemming from the aberrancy of inflammation (1). Therefore, the eventual successful treatment of SLE would probably lie in the integration of various modification techniques. AI can streamline this process by predicting how different modifications (e.g., culture conditions, cytokines, small molecules, or gene-editing tools) will influence the phenotype and behaviour of MSCs (97). AI algorithms can also anticipate how CRISPR-induced gene modifications will affect MSCs, minimising off-target effects, and thus improving the precision of gene editing (148). Similarly, AI can be used to predict how new biologics or drug compounds will alter MSC function, allowing for the creation of more potent and stable therapeutic cells tailored to SLE patients (119).

### Optimising MSC culture methods to lower costs

A major hurdle in MSC therapy is the high cost of cell culture, either by wasting culture medium in 2D cultures or needing a high-cost setup in 3D cultures or large-scale bioreactor conditions (187). AI-driven models can help refine culture conditions by predicting which nutrients, growth factors, and environmental conditions will support the optimal growth and differentiation of MSCs. These models can potentially help identify specific nutrient combinations and conditions that would best encourage MSC differentiation into desired phenotypes, such as those with enhanced immunomodulatory or anti-inflammatory properties, or even into specific cell types such as nerve or sciatic cells (52). Additionally, AI can predict the longevity of MSCs in culture and provide solutions for extending their viability without compromising quality, thus reducing overall production costs (156).

### Precision medicine for SLE patients

AI analyses copious amounts of data, forming correlations and associations within the data and infers changes within the general picture if specific factors change (110, 114). Being a predictive model, it can ‘divinate’ the outcome of taking a specific action. Therefore, it has the potential to revolutionise precision medicine in SLE by analysing patient-specific data (genomic, proteomic, and clinical data) to customise MSC treatments (97). AI models can predict how individual patients will respond to different MSC modifications, helping to create personalised therapies that take their unique immune system profiles and disease characteristics into consideration. Moreover, AI could be utilised to optimise medication doses for SLE patients depending on their patient-specific history, such as metabolism, pharmacogenetics and treatment history (188). By integrating patient-specific data, AI could also potentially forecast the most effective MSC intervention strategies, including the optimal dosage, timing, and possible combination of MSC modifications, enhancing the likelihood of successful treatment outcomes.

### Predicting patient prognosis and treatment success

AI could also be potentially utilised to predict the prognosis of SLE patients and the potential success of MSC-based interventions. By analysing historical data on patient outcomes, AI could identify correlations and associations that suggest which patients are most likely to benefit from MSC therapy (114). This can guide more timely and targeted interventions, improving overall patient care. Other interventions can also be suggested in the event of low efficacy of the present MSC treatment, such as the focus on specific immunosuppressants that cause fewer side effects to the patient (122).

### Predicting the tolerability of immunosuppressants

Immunosuppressants are often essential for treating SLE, but their interactions with MSCs and individual patient biology need to be carefully managed. AI can predict the tolerability of these drugs in both patients and modified MSCs by analysing patient-specific immune profiles (genomics, proteomics and clinical data) and MSC characteristics (189–191). This approach would allow for better decision-making when choosing immunosuppressive treatments that complement MSC therapy, thereby reducing the risk of adverse reactions and improving overall treatment efficacy.

### Discovering novel biologics for MSC modification

AI can be instrumental in identifying new biologics to modify MSCs for SLE treatment. By screening large datasets (especially on gene expression profiling, gene expression network and interaction of biologics with MSC), AI can predict how novel compounds will enhance MSC properties, such as their immunosuppressive or regenerative capabilities (192, 193). This could accelerate the discovery of innovative biologics that are more effective than current options, leading to the development of next-generation MSC therapies. It is also possible that animal biologics could be more effective in modifying MSCs to better suit SLE treatment, and this can only be known by training AI with a proteomics model of known biologics used to modify MSCs.

### Vaccine against Epstein-Barr Virus

The Epstein-Barr Virus (EBV), a member of the herpesvirus family, has been strongly implicated in the pathogenesis of SLE through molecular mimicry, whereby viral antigens resemble host proteins, leading to misdirected immune responses that exacerbate autoimmunity. Numerous studies have demonstrated a strong correlation between EBV infection and SLE, with elevated anti-EBV antibody levels consistently observed in SLE patients compared to healthy controls (22). These findings suggest that prior exposure to EBV may increase the risk of developing SLE, particularly in genetically predisposed individuals (22).

Given this association, the development of an effective EBV vaccine holds significant potential for preventing or mitigating SLE by reducing primary EBV infection or subsequent viral reactivation in high risk populations. Advances in mRNA vaccine platforms, supported by AI and machine learning, are accelerating this effort. AI-driven models can optimise antigen delivery and machine learning, and dosing strategies, while minimising toxicity and enhancing durable activation against EBV (49).

A safe and effective EBV vaccine could reduce autoimmune responses triggered by viral reactivation, thereby lowering disease risk in genetically predisposed individuals. Encouragingly, clinical trials for EBV vaccines are already underway (248), marking a critical step toward preventive strategies that may also benefit patients at risk of EBV-associated autoimmune diseases such as SLE.

## Conclusion

Incorporating AI into MSC research and therapy development for SLE holds transformative potential for disease therapy. AI’s ability to optimise MSC modification, predict patient responses and integrate traditional treatments pave way for more cost-effective, personalised and successful interventions. These advancements might potentially find their way to permanently cure SLE, instead of controlled remission. If such an idea were to be pursued, worldwide collaboration would be necessary, as SLE is a highly complex disease; thus, an enormous dataset would likely be required.

On a basis of cooperative worldwide collaboration, several concrete steps can make this reality. Firstly, establishing a prospective registry of MSC lots that links donor, disease and culture variables with clinical outcomes, creating a shared data infrastructure. Advanced imaging of MSC morphology can be incorporated, relating it to function. With interpretation via machine learning approaches, potency signatures could be established before infusion. This gives confidence that the MSCs used are high potency.

Secondly, flare-prediction models should embed PROMs and wearable data into flare-prediction models. This enables therapy to adapt to the patient, rather than the reverse. Fusing subjective reports of fatigue, pain and function with continuous physiological readouts (i.e. heart rate variability/sleep cycles) allow AI systems to detect early warning signs of flares, which allows tailored and timely interventions.

Thirdly, AI should be trained on MSC modification data to compare priming approaches and gene edits structurally. By training optimisation algorithms on large *in-vitro* datasets, the field could prioritise modifications that reliably enhance immunoregulation while minimising safety risks, accelerating the design of next-generation MSCs. Clinical evaluation should also move toward small, adaptive trials that test dose and patient-matching strategies. Rather than relying solely on large, fixed protocols, Bayesian or response-adaptive designs would allow dosing schedules and subgroup inclusion to evolve in real time based on emerging outcomes, shortening the path to clinically actionable insights.

Finally, these efforts must be anchored by harmonised quality-control benchmarks. Standardised criteria for MSC identity, potency, and genomic integrity, augmented by AI-based analytics, would make results across centres and trials comparable, strengthen regulatory confidence, and provide a common language for the field. Together, these steps delineate a pragmatic agenda for advancing MSC therapy in SLE. By uniting registry science, patient-centred metrics, AI-driven optimisation, adaptive clinical design, and harmonised quality standards, the field can move beyond proof-of-concept to reproducible, personalised, and ethically responsible care. The opportunity now is not simply to test MSCs, but to build the translational ecosystem that will allow them to fulfil their promise for patients with SLE. It is our most sincere hope that this idea can one day be a reality in the medical setting, not just for SLE but for other incurable diseases such as cancer and HIV.

## Supplementary material

Supplementary Table 1 Detailed breakdown of successful MSC modifications with the potential to optimize SLE treatment.

Supplementary Table 2 Detailed breakdown of types of genetic modification for MSCs and targeted genes.
