# Supplementary material for: How artificial intelligence can enable personalized mesenchymal stem cell–based therapeutic strategies in systemic lupus erythematosus
Source: Front Immunol. 2025 Nov 26;16:1654117. doi: 10.3389/fimmu.2025.1654117 (PMC12689592; doi:10.3389/fimmu.2025.1654117)
Supplement: Supplementary Table 1 — Detailed breakdown of successful MSC modifications with the potential to optimize SLE treatment. [file Table1.docx]

**Supplementary materials**

**Supplementary Table 1: Detailed breakdown of successful MSC modifications with the potential to optimize SLE treatment.**
MSCs, mesenchymal stromal cells; SLE, systemic lupus erythematosus; IDO, indoleamine 2,3-dioxygenase; HPL, human platelet lysate

| **Type of MSC modification** | **Example** | **Study(s) type** | **Diseases treated** | **Reference** |
| --- | --- | --- | --- | --- |
| 3-dimensional culture of MSCs | Culturing of MSCs in spheroidal forms - led to increased treatment efficacies due to increased intercellular communication | In vivo (human, mice, rabbit) In vitro | Various: bone, kidney-related, diabetes | [1] |
| Priming or pre-treatment with different biologics | Culturing in HPL - stimulates proliferation and expansion of adipose tissue-derived MSCs | In vitro; gene expression analysis | None | [2] |
|  | Pre-treatment of MSCs with IFN-$\gamma$  - improved IDO and inhibited the production of antibodies | In vivo (SCID-mice) | SLE | [3] |
|  | Pre-treatment with BME, RA, with a mixture of FSK, bFGF, PDGF and HRG, then transplantation of MSCs in vivo post-differentiation  - MSCs differentiated into Schwann cell characteristics can elicit peripheral nervous system regeneration in adult rats | In vivo (rats) | Cut Sciatic nerve | [4] |
|  | Pre-treatment of MSCs with miR-9-5p or TNF-α  - Increased migration rate across injury sites | In vitro | Intimal hyperplasia | [5, 6] |
| Genetic modification and engineering | Transplantation of IL-37  - Prolonged the survival of MSCs, accompanied by symptomatic reduction | In vivo (rats) In vitro | SLE | [7] |
|  | Overexpression of ERBB4/FGF receptors/CBX4  - Improve lifespan of MSCs in vivo, and enhance therapeutics | In vitro In vivo (mice) | Osteoarthritis | [8-10] |
|  | Increased expression of CCR1, CCL2, CXCR2, CXCR4  - Promote therapeutics of MSCs | In vivo (Mice, humans) | Lupus nephritis, Birth Injury-Induced Stress Urinary Incontinence | [11, 12] |
|  | Modified MSCs with biomimetic extracellular matrices & poly (dimethyl siloxane)/ Simultaneous overexpression of CCR1, CXCR2, CXCR4  - Increase MSC migration rate, increase secretion of anti-inflammatory cytokines, accelerate tissue healing | In vivo (mice) | Oral mucositis, Lung injury, Urinary incontinence | [11, 13, 14] |
| Combination therapy with immunosuppressive drugs | Pre-treatment of MSCs with hypoxia/rapamycin & Dickkopf-1  - Maintain MSC stemness, reduce expression of senescence-associated β-gal, and maintain the stemness | In vitro  In vivo (mice) | Liver disease | [15] |
|  | MSCs administered together with either cyclosporin A, mycophenolate mofetil, rapamycin, prednisone or dexamethasone - suppression of pro-inflammatory T cells  - promote activation and function of anti-inflammatory Treg cells  - prolong survival of MSCs in vivo and delay senescence | In vivo (mice) | Transplantation | [16, 17] |
|  | MSCs in combination with prednisone/MMF - Show superior treatment compared to singular therapy, decrease autoantibody levels and inflammatory cytokines in serum, reduce inflammatory cell infiltration in kidney and spleen | In vivo (lupus-prone MRL/lpr mice) | Lupus | [18] |
|  | Administration of dexamethasone and tacrolimus with MSC  - increased MSC persistence, engraftment | In vivo (human stem cells into mice model) | None | [19] |

1. Hazrati, A., et al., *Mesenchymal stromal/stem cells spheroid culture effect on the therapeutic efficacy of these cells and their exosomes: A new strategy to overcome cell therapy limitations.* Biomedicine & Pharmacotherapy, 2022. **152**: p. 113211.

2. Hemeda, H., B. Giebel, and W. Wagner, *Evaluation of human platelet lysate versus fetal bovine serum for culture of mesenchymal stromal cells.* Cytotherapy, 2014. **16**(2): p. 170-180.

3. Kim, D.S., et al., *Enhanced Immunosuppressive Properties of Human Mesenchymal Stem Cells Primed by Interferon-&#x3b3.* eBioMedicine, 2018. **28**: p. 261-273.

4. Dezawa, M., et al., *Sciatic nerve regeneration in rats induced by transplantation of in vitro differentiated bone-marrow stromal cells.* European Journal of Neuroscience, 2001. **14**(11): p. 1771-1776.

5. Bai, X., et al., *TNF-α promotes survival and migration of MSCs under oxidative stress via NF-κB pathway to attenuate intimal hyperplasia in vein grafts.* Journal of Cellular and Molecular Medicine, 2017. **21**(9): p. 2077-2091.

6. Li, X., et al., *MiR-9-5p promotes MSC migration by activating β-catenin signaling pathway.* American Journal of Physiology-Cell Physiology, 2017. **313**(1): p. C80-C93.

7. Xu, J., et al., *Additive Therapeutic Effects of Mesenchymal Stem Cells and IL-37 for Systemic Lupus Erythematosus.* Journal of the American Society of Nephrology, 2020. **31**(1): p. 54-65.

8. Liang, X., et al., *Overexpression of ERBB4 rejuvenates aged mesenchymal stem cells and enhances angiogenesis via PI3K/AKT and MAPK/ERK pathways.* The FASEB Journal, 2019. **33**(3): p. 4559-4570.

9. Coutu, D.L., M. François, and J. Galipeau, *Inhibition of cellular senescence by developmentally regulated FGF receptors in mesenchymal stem cells.* Blood, 2011. **117**(25): p. 6801-6812.

10. Ren, X., et al., *Maintenance of Nucleolar Homeostasis by CBX4 Alleviates Senescence and Osteoarthritis.* Cell Reports, 2019. **26**(13): p. 3643-3656.e7.

11. Jiang, H.-H., et al., *Combined Treatment With CCR1-Overexpressing Mesenchymal Stem Cells and CCL7 Enhances Engraftment and Promotes the Recovery of Simulated Birth Injury-Induced Stress Urinary Incontinence in Rats.* Frontiers in Surgery, 2020. **7**.

12. Che, N., et al., *Impaired B Cell Inhibition by Lupus Bone Marrow Mesenchymal Stem Cells Is Caused by Reduced CCL2 Expression.* The Journal of Immunology, 2014. **193**(10): p. 5306-5314.

13. Shen, Z., et al., *Genetic modification to induce CXCR2 overexpression in mesenchymal stem cells enhances treatment benefits in radiation-induced oral mucositis.* Cell Death & Disease, 2018. **9**(2): p. 229.

14. Zhang, C., et al., *CXCR4-Overexpressing Umbilical Cord Mesenchymal Stem Cells Enhance Protection against Radiation-Induced Lung Injury.* Stem Cells International, 2019. **2019**(1): p. 2457082.

15. Hu, C., Z. Wu, and L. Li, *Pre-treatments enhance the therapeutic effects of mesenchymal stem cells in liver diseases.* J Cell Mol Med, 2020. **24**(1): p. 40-49.

16. Hajkova, M., et al., *Mesenchymal Stem Cells Attenuate the Adverse Effects of Immunosuppressive Drugs on Distinct T Cell Subopulations.* Stem Cell Reviews and Reports, 2017. **13**(1): p. 104-115.

17. Hajkova, M., et al., *Cyclosporine A promotes the therapeutic effect of mesenchymal stem cells on transplantation reaction.* Clin Sci (Lond), 2019. **133**(21): p. 2143-2157.

18. Lee, H.K., et al., *Effect of a Combination of Prednisone or Mycophenolate Mofetil and Mesenchymal Stem Cells on Lupus Symptoms in MRL.Faslpr Mice.* Stem Cells International, 2018. **2018**(1): p. 4273107.

19. Hwang, J.W., et al., *Immunosuppressant Drugs Mitigate Immune Responses Generated by Human Mesenchymal Stem Cells Transplanted into the Mouse Parenchyma.* Cell Transplantation, 2021. **30**: p. 09636897211019025.
