# Supplementary material for: How artificial intelligence can enable personalized mesenchymal stem cell–based therapeutic strategies in systemic lupus erythematosus
Source: Front Immunol. 2025 Nov 26;16:1654117. doi: 10.3389/fimmu.2025.1654117 (PMC12689592; doi:10.3389/fimmu.2025.1654117)
Supplement: Supplementary Table 2 — Detailed breakdown of types of genetic modification for MSCs and targeted genes. [file Table2.docx]

**Supplementary Table 2: Detailed Breakdown of Types of Genetic Modification for MSCs and Targeted Genes**

| Type of modifications | Pros | Cons | MSC source | Genes modified | References |
| --- | --- | --- | --- | --- | --- |
| Lentivirus | Transduction of dividing and non-dividing cells  Stable and efficient gene transfer  High levels of transgene expression  High Titters | Risk of insertional mutagenesis  Limited by the size of the therapeutic gene insert | Bone marrow | miRs | [1] |
|  |  |  |  | Periostin | [2] |
|  |  |  |  | PEDF | [3] |
|  |  |  |  | ROR2 | [4] |
|  |  |  |  | SDF-1 | [5] |
|  |  |  |  | TGF-b1 | [6] |
|  |  |  |  | β-catenin | [7] |
|  |  |  | Adipose tissue | Neuregulin4 (Nrg4) | [8] |
|  |  |  |  | Telomerase  (TERT) | [8] |
|  |  |  |  | Myocardin  (MYOCD) | [9] |
|  |  |  | Human umbilical cord | TIMP2 | [10] |
|  |  |  | Human Amniotic fluid | Nrf2 | [11] |
| Adenovirus | Efficient gene transfer  Large insert capacity | Transient gene expression  High immunogenicity | Bone marrows | Integrin-linked kinase(ILK) | [12] |
|  |  |  | Adipose tissue | Neuregulin 1 (NRG1) | [13] |
|  |  |  | Human umbilical cord blood | Urokinase plasminogen activator | [14] |
|  |  |  | Human umbilical cord | Thioredoxin 1 (Trx-1) | [15] |
| Retrovirus | Easy manipulation and vector design  Stable and efficient gene transfer  Extensive cell transduction tropism | Only transduce dividing cells  Risk of insertional mutagenesis | Bone marrow | HGF | [16] |
|  |  |  | Adipose tissue | TNF-alpha | [17] |
| Plasmid transfection | Low immunogenicity  Safe manipulation | Low transfection efficiency  Transient gene expression | Bone marrow | miR-16-5p | [18] |
|  |  |  | Adipose tissue | Oct4 and Sox2 | [19] |
|  |  |  | Human umbilical cord blood | Stromal cell derived factor-1[20] | [20] |
| Zinc Finger nuclease (ZFN) | Precise gene edits (mutation, insertion, replacement, deletion)  Relative high efficiency | Off-target risks  Complicated experimental design  Difficult to target non-G-rich sequences | Bone marrow | CCR5 | [21] |
| TALEN | Precise gene edits  Relative high efficiency | Off-target risk  Complicated experimental design  50 targeted bases must be ‘T’ for each TALEN monomer | Amniotic fluid | IL-10 | [22] |
| CRISPR/  Cas9 | Precise gene edits  Relative high efficiency  High ease of multiplexing | Off-target risk  Target sequencing must precede a protospacer adjacent motif (PAM) | Immortalised MSC cell line | Runx2 and SOX9 | [23] |
|  |  |  | Bone marrow | Platelet-derived growth factor B(PDGF-B) | [24] |
|  |  |  | Adipose tissue | Thymidine kinase2 (TK2) | [25] |

PAM, protospacer adjacent motif; MSC, mesenchymal stromal cells; TK2, Thymidine kinase 2

1. Shahror, R.A., et al., *Transplantation of Mesenchymal Stem Cells Overexpressing Fibroblast Growth Factor 21 Facilitates Cognitive Recovery and Enhances Neurogenesis in a Mouse Model of Traumatic Brain Injury.* Journal of Neurotrauma, 2019. **37**(1): p. 14-26.

2. Cho, Y.-H., et al., *Enhancement of MSC adhesion and therapeutic efficiency in ischemic heart using lentivirus delivery with periostin.* Biomaterials, 2012. **33**(5): p. 1376-1385.

3. Gao, Y., et al., *Human mesenchymal stem cells overexpressing pigment epithelium-derived factor inhibit hepatocellular carcinoma in nude mice.* Oncogene, 2010. **29**(19): p. 2784-2794.

4. Cai, S.-x., et al., *The orphan receptor tyrosine kinase ROR2 facilitates MSCs to repair lung injury in ARDS animal model.* Cell Transplantation, 2016. **25**(8): p. 1561-1574.

5. Kizilay Mancini, Ö., et al., *A proinflammatory secretome mediates the impaired immunopotency of human mesenchymal stromal cells in elderly patients with atherosclerosis.* Stem cells translational medicine, 2017. **6**(4): p. 1132-1140.

6. Fierro, F.A., et al., *Effects on proliferation and differentiation of multipotent bone marrow stromal cells engineered to express growth factors for combined cell and gene therapy.* Stem cells, 2011. **29**(11): p. 1727-1737.

7. Cai, S.-x., et al., *Activation of Wnt/β-catenin signalling promotes mesenchymal stem cells to repair injured alveolar epithelium induced by lipopolysaccharide in mice.* Stem cell research & therapy, 2015. **6**: p. 1-11.

8. Xiao, X., Z.-C. Wu, and K.-C. Chou, *A multi-label classifier for predicting the subcellular localization of gram-negative bacterial proteins with both single and multiple sites.* PloS one, 2011. **6**(6): p. e20592.

9. Madonna, R., et al., *Proteomic analysis of the secretome of adipose tissue-derived murine mesenchymal cells overexpressing telomerase and myocardin.* Journal of Molecular and Cellular Cardiology, 2019. **131**: p. 171-186.

10. Ni, J., et al., *Exosomes derived from TIMP2‐modified human umbilical cord mesenchymal stem cells enhance the repair effect in rat model with myocardial infarction possibly by the Akt/Sfrp2 pathway.* Oxidative Medicine and Cellular Longevity, 2019. **2019**(1): p. 1958941.

11. Lim, R.B.T., et al., *Gender and ethnic differences in incidence and survival of lymphoid neoplasm subtypes in an A sian population: Secular trends of a population‐based cancer registry from 1998 to 2012.* International Journal of Cancer, 2015. **137**(11): p. 2674-2687.

12. Mao, Q., et al., *Mesenchymal stem cells overexpressing integrin-linked kinase attenuate left ventricular remodeling and improve cardiac function after myocardial infarction.* Molecular and Cellular Biochemistry, 2014. **397**: p. 203-214.

13. Ryu, S., et al., *Therapeutic efficacy of neuregulin 1-expressing human adipose-derived mesenchymal stem cells for ischemic stroke.* PloS one, 2019. **14**(9): p. e0222587.

14. Pulukuri, S.M.K., et al., *Epigenetic upregulation of urokinase plasminogen activator promotes the tropism of mesenchymal stem cells for tumor cells.* Molecular Cancer Research, 2010. **8**(8): p. 1074-1083.

15. Hu, J., et al., *Infusion of Trx-1-overexpressing hucMSC prolongs the survival of acutely irradiated NOD/SCID mice by decreasing excessive inflammatory injury.* PLoS One, 2013. **8**(11): p. e78227.

16. Song, Y.S., et al., *Mesenchymal stem cells overexpressing hepatocyte growth factor (HGF) inhibit collagen deposit and improve bladder function in rat model of bladder outlet obstruction.* Cell Transplantation, 2012. **21**(8): p. 1641-1650.

17. Tyciakova, S., et al., *Mesenchymal stromal cells producing TNFα lack inhibitory effect against A375 experimental lung metastases.* Neoplasma, 2017. **64**(2): p. 222-227.

18. Xu, Y., et al., *microRNA‐16‐5p‐containing exosomes derived from bone marrow‐derived mesenchymal stem cells inhibit proliferation, migration, and invasion, while promoting apoptosis of colorectal cancer cells by downregulating ITGA2.* Journal of cellular physiology, 2019. **234**(11): p. 21380-21394.

19. Han, S.-M., et al., *Enhanced proliferation and differentiation of Oct4-and Sox2-overexpressing human adipose tissue mesenchymal stem cells.* Experimental & molecular medicine, 2014. **46**(6): p. e101-e101.

20. Unzek, S., et al., *SDF-1 recruits cardiac stem cell-like cells that depolarize in vivo.* Cell transplantation, 2007. **16**(9): p. 879-886.

21. Manotham, K., S. Chattong, and A. Setpakdee, *Generation of CCR5-defective CD34 cells from ZFN-driven stop codon-integrated mesenchymal stem cell clones.* Journal of Biomedical Science, 2015. **22**: p. 1-8.

22. Meng, D., et al., *Interleukin 10-secreting MSCs via TALEN-mediated gene editing attenuates left ventricular remodeling after myocardial infarction.* Cell Physiol Biochem, 2019. **52**(4): p. 728-741.

23. Carstairs, A., *Development of in vitro skeletal disease models using CRISPR/Cas9 genome editing in immortalised mesenchymal stem cells*. 2017, University of York.

24. Kosaric, N., et al., *Human Mesenchymal stromal cells engineered to overexpress PDGF-B using CRISPR/Cas9/rAAV6-based tools improve wound healing.* Plastic and Reconstructive Surgery–Global Open, 2017. **5**(4S): p. 74.

25. Provenzano, C., et al., *CRISPR/Cas9-mediated deletion of CTG expansions recovers normal phenotype in myogenic cells derived from myotonic dystrophy 1 patients.* Molecular Therapy-Nucleic Acids, 2017. **9**: p. 337-348.
